# Discovery of cyanopyridine scaffold as novel indoleamine-2,3-dioxygenase 1 (IDO1) inhibitors through virtual screening and preliminary hit optimisation

**DOI:** 10.1080/14756366.2018.1480614

**Published:** 2019-01-02

**Authors:** Xi Xu, Jie Ren, Yinghe Ma, Hongting Liu, Quanjin Rong, Yifan Feng, Yameng Wang, Yu Cheng, Ruijia Ge, Zhiyu Li, Jinlei Bian

**Affiliations:** a State Key Laboratory of Natural Medicines, Jiangsu Key Laboratory of Drug Design and Optimization, China Pharmaceutical University, Nanjing, People’s Republic of China;; b Department of Medicinal Chemistry, School of Pharmacy, China Pharmaceutical University, Nanjing, People’s Republic of China;; c The Madeira School, McLean, VA, USA

**Keywords:** Indoleamine 2,3-dioxygenase 1, inhibitor, virtual screen, cyanopyridine scaffold, hit optimisation

## Abstract

With the aim of discovering novel IDO1 inhibitors, a combined similarity search and molecular docking approach was employed to the discovery of 32 hit compounds. Testing the screened hit compounds has led to several novel submicromolar inhibitors. Especially for compounds **LVS-019** with cyanopyridine scaffold, showed good IDO1 inhibitory activity. To discover more compounds with similar structures to **LVS-019**, a shape-based model was then generated on the basis of it and the second-round virtual screening was carried out leading to 23 derivatives. Molecular docking studies suggested a possible binding mode of **LVS-019**, which provides a good starting point for the development of cyanopyridine scaffold compounds as potent IDO1 inhibitor. To improve potency of these hits, we further designed and synthesised another 14 derivatives of **LVS-019**. Among these compounds, **LBJ-10** showed improved potency compared to the hits and displayed comparable potency to the control GDC-0919 analogue. **LBJ-10** can serve as ideal leads for further modifications as IDO1 inhibitors for cancer treatment.

## Introduction

**Figure 1.**
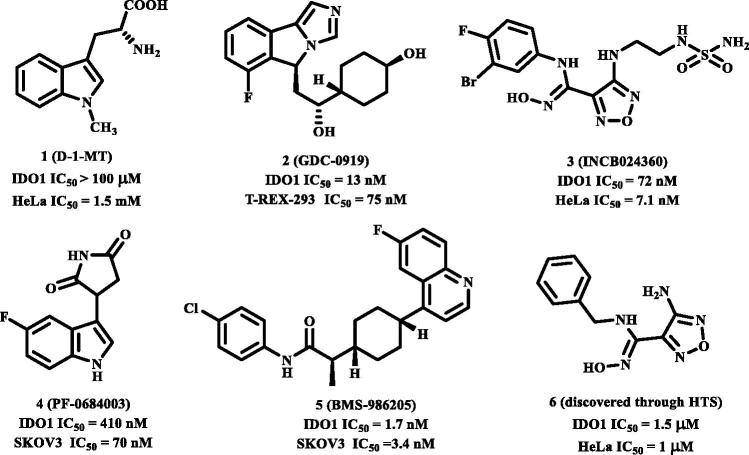
The structures of clinical IDO1 inhibitors (1–5) and hit compound (6) of INCB024360.

Indoleamine 2,3-dioxygenase 1 (IDO1) is a haeme-containing enzyme that catalyses the first and rate limiting step of *L*-tryptophan conversion to *N*-kynurenine along the kynurenine pathway^1^^,^^2^. IDO1 is a monomeric enzyme, which degrades different indoleamine substrates, including *L*-Trp, *D*-Trp, and tryptamine, and is ubiquitously expressed in many tissues^3^. The expression of IDO1 could be induced by interferon-γ, lipopolysaccharide, and tumour necrosis factor^4–6^. Thus, within the immune system IDO1 gets highly up-regulated in response to inflammatory signals under pathophysiological conditions. Over-expression of IDO1 corrects with poor prognosis in different types of cancers, including melanoma, pancreatic, ovarian, colorectal, and others^7^. It is believed that IDO1 could subvert the immunomodulatory mechanism and promote tolerance local to the cancer^7^^,^^8^. Recently, multiple studies, including si-RNA, IDO1 null mice, and small-molecule inhibitors, have appeared validating IDO1 as a therapeutic target for cancer immunotherapy^9–14^. In addition, increased IDO1 expression correlates with different tumour progression parameters and shorter patient survival has also been discovered^15^. IDO1 inhibitors have a synergistic effect when used in combination with anti-PD1 mAbs^16^ and some other anticancer drugs, such as cyclophosphamide, doxorubicin, paclitaxel, and cisplatin^17^. All this evidence confirms that IDO1 inhibition might enhance the efficacy of pharmacological cancer therapy and is a potential breakthrough approach to cancer therapy. As such, the enzyme IDO1 has emerged as a promising therapeutic target, prompting a number of potent IDO1 inhibitors have been identified recently. For the past few years, we have witnessed many efforts been focussed on discovering potential IDO1 inhibitors and hundreds of compounds have been reported according to the scientific and patent literature. However, to the best of our knowledge, only five small-molecule compounds are currently undergoing an extensive development programme (either alone or in combination) and no drug has been approved by FDA up to now^18^. Among these IDO1 inhibitors in clinical, 1-methyl-*D*-tryptophan (*D*-1-MT, indoximod, **1**, Figure 1) is developed by NewLink Genetics, and currently is undergoing evaluation for multiple human clinical trials^12^^,^^19–22^. NewLink Genetics developed another IDO1 inhibitor NLG-919 (also named GDC-0919, **2**) which currently is in phase I clinical trials in subjects with recurrent advanced solid tumours^23^^,^^24^. Incyte Corporation developed a clinical candidate hydroxyamidine INCB024360 (named Epacadostat, **3**), which was in phase II/III clinical trials, used as a monotherapy as well as in combination use with various antibodies, for the treatment of advanced or metastatic cancers^25^. Recently, five latest IDO1 inhibitors have also entered clinical trials within the last few months. Pfizer Inc./iTeos Therapeutics SA reported PF-06840003 (**4**), which is under phase I clinical trials for the treatment of patients with grade IV glioblastoma or grade III anaplastic glioma^26^^,^^27^. Bristol-Myers Squibb Corporation reported the other latest IDO1 inhibitor BMS-986205 (ONO-7701, **5**), which is in phase I/II clinical trials in combination with nivolumab in patients with advanced cancers^28^. JiangSu Hengrui Medicine Corporation developed a small-molecule IDO1/TDO dual inhibitor **SHR-9146** (NCT03208959, HTI-1090) with favourable preclinical oral bioavailability and safety profiles, which is under phase I clinical trials in subjects with advanced solid tumours. A novel prodrug of indoximod (named NLG-802 or NCT03164603) with enhanced pharmacokinetic properties was also entered into Phase I study recently by NewLink Genetics. The structures of **SHR-9146** and NLG-802 have not been disclosed until now.

**Figure 2.**
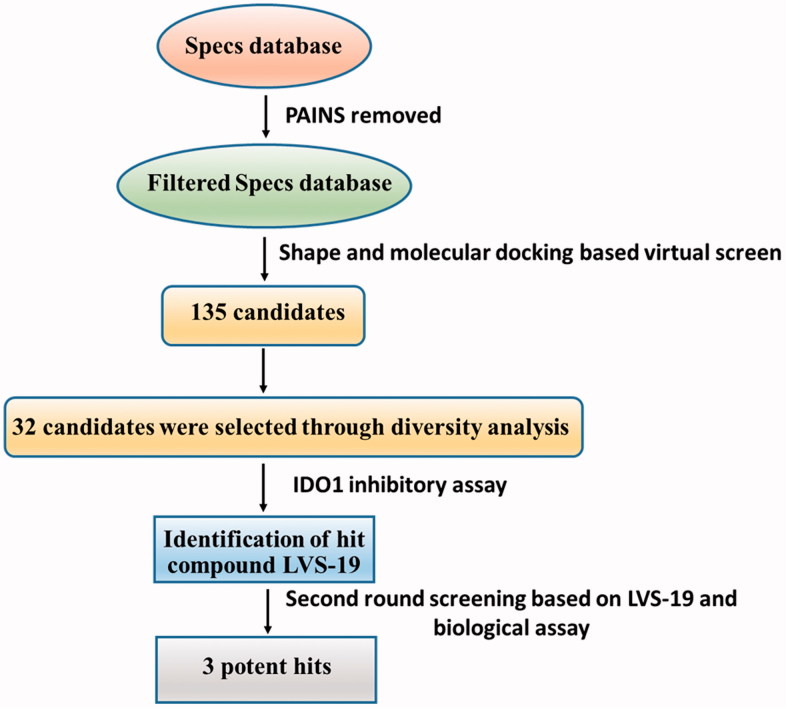
Shows the computational protocol that was applied to IDO1 inhibitors. Full details of each step are discussed in the following paragraphs.

With only these IDO1 inhibitors under clinical trials and no drug has been approved, discovering novel IDO1 inhibitors suitable for development into clinical candidates is still in great demand. Further exploring these IDO1 inhibitors, we found that many of them with new structural scaffolds were discovered by structure-based design, high-throughput screening, and natural product derivatisation^29^. Notably, the lead compound 4-amino-1,2,5-oxadiazole-3-carboximidamide (**6**) of currently most promising IDO1 inhibitor INCB024360 was discovered through high-throughput screening (HTS) of Incyte’s corporate collection^13^. It indicated that HTS is an efficient method to discover novel IDO1 inhibitors. As a complementary approach to experimental HTS, virtual screening (VS) recently has drawn lots of attention for saving time and expenses^30^. Similarity search and molecular docking are two most commonly used methods in the discovery of small-molecule inhibitors. However, independent VS methods are facing the insufficient accuracy of scoring function which remains the bottleneck of VS^31^^,^^32^. Thus, in this study, using the combined similarity search and molecular docking approaches, we developed a VS strategy to discover new inhibitors targeting IDO1. The selected molecules were further evaluated by biochemical analysis, and the predicted binding modes of potent inhibitors were analysed. Among the potent novel molecules, a cyanopyridine scaffold named **LVS-19** attracted us the most. We then selected **LVS-19** to generate the rapid overlay of chemical structures (ROCS) model. With strictly chemical similarity analysis, a second-round shape-based screening was performed. Twenty-three derivatives **LVS-33∼LVS-56** were finally retained and purchased from Specs database. They were also evaluated for their IDO1 inhibitory activity. A schematic of the overall VS protocol is presented in Figure 2. In addition, to improve potency of these hit compounds, another 14 derivatives (**LBJ-01∼LBJ-14**) of **LVS-019** were designed and synthesised through rational drug designation.

## Materials and methods

### Virtual screening

#### Computational calculation

The Specs database file (version 01/2012) was downloaded from the official Web site (www.specs.net) and these compounds were prepared with the module of Search Conformations in MOE2016.09^33^ to provide an average of 140 conformations. These structures were washed, i.e. all inorganic compounds were removed and all ionisable groups were set to coordinated with neutral pH conditions. Energetically minimised conformations were generated using the MMFF94x force field^34^.

#### Creation of the ROCS model and the virtual screens based on it

The shape-based pharmacophore model in VS was generated by employing ROCS^35^. The algorithm is based on the idea that the molecular shape of compounds is similar if the molecules overlay well and any volume mismatch results from shape dissimilarity. VSs were then performed based on the query models of INCB024360, GDC-0919, Amg-1, and **LVS-019**. The parameters for the ROCS run were set as follows: rankby = combo and besthits =1. The screening score for a particular database compounds was set to the maximum combo score between the database compound and model compounds.

#### Molecular docking

##### Native-docking

The crystallographic coordinates of human IDO1-inhibitors (PDB code 2D0T, 4PK5, 5EK2, 5EK3, 5EK4, and 5ETW) were used to conduct native-docking^36–38^ (When we have finished our works, some other crystallographic coordinates are available. We added the newly reported PDB information of these structures in the Supporting Information.) These ligands were docked back into their corresponding protein structures using Gold5.1 (CCDCs software GOLD version 5.1)^39^^,^^40^, CDOCKER (DS 4.0)^41^, alpha PMI (Principal Moments of Inertia, MOE 2016.09)^42^, AutoDock^43^, and Glide (Schrödinger 2009)^44^. To define the binding pocket of IDO1 cocrystal structure, residues around the native ligand (radius set as 8.0 Å) were selected for molecular docking. All other parameters are set as default. The docking results were evaluated through comparison of the best docked poses and the real cocrystallised pose to measure docking reliability. The best docking pose was defined as that top-scoring poses. The docking software was evaluated with root mean square deviation (RMSD) value.

##### Cross-docking

The complexes used in native-docking were also selected to perform cross-docking evaluation. After superposing the crystallographic complexes, the native ligands were extracted, merged in a unique database and docked into each protein structure with five docking combination protocols used in native-docking. For each of the five docking protocols and six PDB structures, a database of five ligands was docked to each of the six protein structures (the self-ligand was excluded for the cross-docking computation to follow a strict cross-docking procedure). Ten poses were generated for each of the ligand–protein couples and the RMSD values between the top-scoring pose and the crystallographic conformation of the ligand were computed. The evaluation of each protein-docking protocol couple was performed considering the RMSD of the top-scoring poses of each of the five native ligands docked on the same protein with the same protocol.

##### Screen with docking

GOLD was a powerful genetic algorithm (GA) method for conformation search and docking. In the present study, the binding site was defined by carefully analysing the protein–ligand interactions between IDO1 and its inhibitor. Residues around the original ligand (radius 8.0 Å) were also included in the active site. All water molecules have been removed from the protein structure. Docking studies were performed using the standard default settings with 10 GA runs on each molecule. For each of the GA runs, a maximum of 125,000 operations were performed. The binding energy of the ligands with protein was predicted using the Goldscore by choosing in the appropriate scoring function goldscore.p450_pdb.params file from those that have been supplied with the GOLD installation^45^. All other parameters were kept at their default values.

### Biological experiments

#### Enzyme-based IDO1 activity assay

The recombinant human IDO1 was expressed and purified as previously reported^38^. The IDO1 activity assay was also carried out as previously described^38^. In brief, the IDO1 activity of test compounds was measured in the assay buffer (100 µL) containing 50 mM potassium phosphate buffer (pH = 6.5), 25 mM neutralised ascorbic acid (10 µL), 10 µM methylene blue (10 µL), 100 µg/mL catalase (10 µL), 200 µM L-tryptophan (10 µL), purified IDO1 enzyme (10 µL, 25 µg/mL). The test compounds (10 µL, in DMSO, 0.5% final concentration) were added in 96-well plates. The reaction was carried out at 37 °C for 1 h. To convert *N*-formylkynurenine to kynurenine, 10 µL of NaOH (1M) was added and incubated at 60 °C for 30 min. Then the mixture was cooled at 0 °C and centrifuged at 10,000 rpm for 10 min. Lastly, 100 µL supernatant form each well was transferred to a new 96-well plate and mixed at equal volume with 2% (*w*/*v*) *p*-dimethylaminobenzaldehyde (*p*-DMAB, Ehrlich's reagent) in acetic acid. The yellow colour generated from the reaction with kynurenine was measured at 480 nm. The percentage of inhibition is reported as (100 − (*A*/*B* × 100))/100, where *A* is the IDO1 activity in the presence of the test compound, and *B* is the IDO1 activity in the absence of the test compound.

#### Cell-based IDO1 activity assay

HeLa cells were seeded in 96-well culture plates at a density of 5 × 10^4^ per well. On the next day, human IFN-γ (20 ng/well) and compounds in a total volume of 200 µL culture medium containing 15 µg/mL of L-tryptophan were added to the cells. After incubation for 24 h, 140 µL of supernatant was mixed with 10 µL of 6.1 N trichloroacetic acid and the mixture was incubated for 30 min at 60 °C. The reaction mixture was then centrifuged for 20 min at 4000 rpm at 0 °C to remove sediments. About 100 µL of the supernatant was mixed with 100 µL of 2% (*w*/*v*) *p*-DMAB in acetic acid and measured at 480 nm. The final DMSO concentration was 0.5%. The data were analysed using Graphpad Prism 6.

#### Measurement of IDO1 absorbance spectra

Absorbance spectra (250–480 nm) were measured immediately after addition of compound **LBJ-10** (20 µM in 0.5% DMSO) to IDO1 (5 µM) in phosphate buffer (pH 6.5) using a Tecan Safire2 microplate reader.

#### Determination of the inhibition type

The standard assay mixture (1 ml) contained 50 mM potassium phosphate buffer (pH = 6.5), 25 mM neutralised ascorbic acid, 10 µM methylene blue, 100 µg/mL catalase, 50 µM L-Trp, DMSO (0.5%), and eight different concentrations of IDO1. In addition, 1 µM **LBJ-10** solution was added into the mixture. The reaction, at 37 °C, was started by the addition of the substrate and terminated after 60 min by adding 200 µL of 30% (*w*/*v*) trichloroacetic acid and further incubated at 50 °C for 30 min to hydrolyse *N*-formylkynurenine to produce kynurenine. After centrifugation at 12,000 rpm for 15 min at 20 °C, kynurenine in the supernatant (1 mL) was measured by a HPLC system with a reversed phase column (4.6 mm × 15 cm) of GL Sciences Inc. The mobile phase was 10 mM ammonium acetate containing 10% (*w*/v) methanol, and kynurenine was detected by absorbance at 360 nm. All determinations were carried out in duplicate. The data presented are average values. Based on the results of the activity assay, a function of rate ([V]) against enzyme amount ([E]) was proposed.

#### Water solubility evaluation

The aqueous solubility was determined on a Gemini Profiler instrument (pION) by the “goldstandard” Avdeef–Bucher potentiometric titration method^46^^,^^47^.

#### Cell viability assay

Growth inhibition was determined by the MTT colorimetric assay. HeLa cells were plated in 96-well plates at a density of 10,000 cells/mL and allowed to attach overnight (16 h). Cells were plated in 96-well plates at a density of 10,000 cells/mL and allowed to attach overnight (16 h). Cells were then exposure to 50 µM of test compounds in triplicate for 48 h at 37 °C under a humidified atmosphere containing 5% CO_2_. MTT (50 µg) was added and the cells were incubated for another 4 h. Medium/MTT solutions were removed carefully by aspiration, the MTT formazan crystals were dissolved in 100 µL of DMSO, and absorbance was determined on a plate reader at 560 nm.

### Chemistry

#### General procedures

All chemicals and reagents were purchased from commercial sources. Organic solutions were concentrated in a rotary evaporator (Bu¨chi Rotavapor) below 55 °C under reduced pressure. Silica gel thin-layer chromatography was performed on precoated plates GF-254 and visualised under UV light. Melting points were determined with a Melt-Temp II apparatus. ^1^H NMR and 13C NMR spectra were recorded on a Bruker AV-300 instrument using deuterated solvents with tetramethylsilane as internal standard. Chemical shifts are given in ppm (*δ*). The multiplicities are denoted as follows: s, singlet; d, doublet; t, triplet; q, quartette; m, multiplet; br s, broad singlet. IR spectra were recorded on a Nicolet iS10 Avatar FT-IR spectrometer using KBr film. ESI-mass and high-resolution mass spectra (HRMS) were recorded on a Water Q-Tofmicro mass spectrometer. Analytical results are within 0.40% of the theoretical values. Purity of the compounds was analysed by HPLC (Agilent Technologies 1260 Infinity) using 80:20 MeOH/H_2_O as the mobile phase with a flow rate of 0.8 ml/min on a C18 column. All tested compounds exhibited greater than 95% purity unless otherwise noted.

#### Synthesis

##### (E)-4-Ethoxy-1,1,1-trifluorobut-3-en-2-one (16)

To the ethoxyethene (7.2 g, 0.1 mol, 1 eq) in pyridine (11.9 g, 0.15 mol, 1.5 eq) was added trifluoroacetic anhydride by dripping slowly at 0 °C. Then stirring for 12 h at room temperature. The mixture was adjusted to neutral with 2 N HCl. The organic was washed with water and brine successively, dried over anhydrous sodium sulphate, and concentrated in vacuum to obtain yellow oily liquid (10.1 g, 60%). HRMS *m*/*z* [M + H]^+^ calculated for C_6_H_8_F_3_O_2_: 169.0471, found: 169.0477.

##### 2-Hydroxy-6-(trifluoromethyl)nicotinonitrile (17)

Intermediate **16** (8.4 g, 0.05 mol, 1 eq) was dissolved in 50 ml of ethanol, and nitrile acetamide (3.5 g, 0.05 mol, 1 eq) was added at 0 °C. Then sodium ethoxide (25%, 6.8 g, 0.1 mol, 2 eq) was slowly added. The mixture was stirred under reflux for 12 h and acidified with 2 N HCl to pH =3–4. Subsequently, the solvent was concentrated in vacuum and diluted with ethyl acetate. The solution was washed with water and brine, dried over anhydrous sodium sulphate, and concentrated in vacuum to obtained the product as a yellow solid (6.1 g, 65%). HRMS *m*/*z* [M + H]^+^ calculated for C_7_H_4_F_3_N_2_O: 189.0270, found: 189.0270.

##### 2-Chloro-6-(trifluoromethyl)nicotinonitrile (18a)

Compound **17** (3.0 g, 15.96 mmol, 1 eq) was added to the bottle, and phosphorus oxychloride (17.0 g, 111.69 mmol, 7 eq) was added. The mixture was refluxed for 2.5 h. Removing excess phosphorus oxychloride under reduced pressure, then poured the residue to the ice water and washed with sodium bicarbonate. The mixture was then extracted twice with AcOEt, washed five times with a saturated solution of NaCl, dried over Na_2_SO_4_ and concentrated. The crude product was purified by flash column chromatograph on silica gel (PE/EA =10:1) to give **18a** in 45% yield as a milk white powder. HRMS *m*/*z* [M + H]^+^ calculated for C_7_H_3_ClF_3_N_2_: 206.9931, found: 206.9933.

##### N-(4-Fluorophenyl)-2-hydroxyacetamide (21a)

To a solution of 4-fluoroaniline (11.1 g, 0.1 mol, 1 eq) at room temperature was added glycolic acid (8.4 g, 0.11 mol, 1.1 eq). The mixture was then stirred at 115 °C for 5.5 h and quenched at room temperature by addition of a saturated solution of sodium bicarbonate then obtain precipitate **21a** (14.4 g, 85%). HRMS *m*/*z* [M + H]^+^ calculated for C_8_H_9_FNO_2_: 170.0612, found: 170.0614.

##### N-(4-Fluorophenyl)-2-mercaptoacetamide (21b)

To a solution of 4-fluoroaniline (11.1 g, 0.1 mol, 1 eq) at room temperature was added mercaptoacetic acid (10.1 g, 0.11 mol, 1.1 eq). The mixture was then stirred at 125 °C for 6 h and quenched at room temperature by addition of a saturated solution of sodium bicarbonate then obtain precipitate **21 b** (16.3 g, 88%). HRMS *m*/*z* [M + H]^+^ calculated for C_8_H_9_FNOS: 186.0383, found: 186.0382.

##### 2-Mercapto-N-phenylacetamide (21c)

To a solution aniline (9.3 g, 0.1 mol, 1 eq) at room temperature was added mercaptoacetic acid (10.1 g, 0.11 mol, 1.1 eq). The mixture was then stirred at 110 °C for 6 h and quenched at room temperature by addition of a saturated solution of sodium bicarbonate then obtain precipitate **21c** (14.0 g, 84%). HRMS *m*/*z* [M + H]^+^ calculated for C_8_H_10_NOS: 168.0478, found: 168.0472.

##### N-(2-Fluorophenyl)-2-mercaptoacetamide (21d)

To a solution 2-fluoroaniline (11.1 g, 0.1 mol, 1 eq) at room temperature was added mercaptoacetic acid (10.1 g, 0.11 mol, 1.1 eq). The mixture was then stirred at 120 °C for 6 h and quenched at room temperature by addition of a saturated solution of sodium bicarbonate then obtain precipitate **21d** (16.1 g, 87%). HRMS *m*/*z* [M + H]^+^ calculated for C_8_H_9_FNOS: 186.0383, found: 186.0386.

##### N-(3-Chloro-4-fluorophenyl)-2-mercaptoacetamide (21e)

To a solution 3-chloro-4-fluoroaniline (14.5 g, 0.1 mol, 1 eq) at room temperature was added mercaptoacetic acid (10.1 g, 0.11 mol, 1.1 eq). The mixture was then stirred at 150 °C for 6 h and quenched at room temperature by addition of a saturated solution of sodium bicarbonate then obtain precipitate **21e** (19.3 g, 88%). HRMS *m*/*z* [M + H]^+^ calculated for C_8_H_8_ClFNOS: 219.9994, found: 220.0000.

##### 2-Mercapto-N-(p-tolyl)acetamide (21f)

To a solution 4-methylaniline (10.7 g, 0.1 mol, 1 eq) at room temperature was added mercaptoacetic acid (10.1 g, 0.11 mol, 1.1 eq). The mixture was then stirred at 105 °C for 6 h and quenched at room temperature by addition of a saturated solution of sodium bicarbonate then obtain precipitate **21f** (15.4 g, 85%). HRMS *m*/*z* [M + H]^+^ calculated for C_9_H_12_NOS: 182.0634, found: 182.0636.

##### 2-Mercapto-N-(4-methoxyphenyl)acetamide (21g)

To a solution 4-methoxyaniline (12.3 g, 0.1 mol, 1 eq) at room temperature was added mercaptoacetic acid (10.1 g, 0.11 mol, 1.1 eq). The mixture was then stirred at 100 °C for 6 h and quenched at room temperature by addition of a saturated solution of sodium bicarbonate then obtain precipitate **21 g** (17.5 g, 89%). HRMS *m*/*z* [M + H]^+^ calculated for C_9_H_12_NO_2_S: 198.0583, found: 198.0586.

##### 2-((3-Cyanopyridin-2-yl)oxy)-N-(4-fluorophenyl)acetamide (LBJ-01)

To a solution of 2-chloro-3-nitrile pyridine (1.4 g, 10 mmol, 1 eq) and **21a** (2.5 g, 15 mmol, 1.5 eq) in anhydrous DMF at room temperature was added K_2_CO_3_ (1.4, 10 mmol, 1 eq). The mixture was then stirred at 20 °C for 15 min, the reaction was quenched with water then the product was obtained by filtration and the precipitate was purified by silica gel chromatography (PE/EA, v/v, 5:1) to give the title product as a white solid (1.2 g, 43%). m.p. 234–236 °C. HRMS *m/z* [M + Na]^+^ calculated for C_14_H_10_FN_3_OSNa: 294.0655, found: 294.0659. ^1^H NMR (300 MHz, DMSO-d_6_) *δ*: 10.27 (s, 1H, -NH-), 8.42 (d, *J* = 3.3 Hz, 1H, -ArH), 8.33 (d, *J* = 6.73 Hz, 1H, -ArH), 7.58 (s, 2H, -ArH), 7.24–7.12 (m, 3H, -ArH), 5.09 (s, 2H, -CH_2_-) ppm. 13C NMR (75 MHz, DMSO-d_6_) *δ*: 165.6, 162.5, 158.0 (^1^*J*_CF_ = 240.0 Hz), 151.5, 144.2, 134.9, 134.8, 120.9 (^3^*J*_CF_ = 8.3 Hz), 117.8, 115.4 (^2^*J*_CF_ = 22.5 Hz), 95.7, 64.7 ppm.

##### 2-((2-Cyanophenyl)thio)-N-(4-fluorophenyl)acetamide (LBJ-02)

The title compound was prepared from 2-chlorobenzonitrile and **21b** following the synthesis route of **LBJ-01**. Yellow solid (1.1 g, 40%). m.p. 215–217 °C. HRMS *m/z* [M + H]^+^ calculated for C_15_H_12_FN_2_OS: 287.0654, found: 287.0668. ^1^H NMR (300 MHz, DMSO-d_6_) *δ*: 9.44 (s, 1H, -NH-), 8.10 (d, *J* = 9.0 Hz, 1H, -ArH), 7.89 (d, *J* = 6.0 Hz, 1H, -ArH), 7.73–7.68 (m, 2H, -ArH), 7.51 (t, *J* = 7.5 Hz, 1H, -ArH), 7.42 (t, *J* = 7.5 Hz, 1H, -ArH), 7.29 (s, 2H, -CH_2_-), 7.16 (t, *J* = 9.0 Hz, 2H, -ArH) ppm. 13C NMR (75 MHz, DMSO-d_6_) *δ*: 164.9, 162.5, 158.0 (^1^*J*_CF_ = 238.6 Hz), 148.0, 144.3, 135.2 121.0 (^3^*J*_CF_ = 7.5 Hz), 116.5, 116.4, 115.2 (^2^*J*_CF_ = 21.8 Hz), 114.5, 109.6, 35.1 ppm.

##### 2-((3-Cyanopyridin-2-yl)thio)-N-(4-fluorophenyl)acetamide (LBJ-03)

The title compound was prepared from 2-chloro-3-cyanopyridine and **21b** following the synthesis route of **LBJ-01**. Yellow solid (1.3 g, 45%). m.p. 198–201 °C. HRMS *m/z* [M + H]^+^ calculated for C_14_H_11_FN_3_OS: 288.0601, found: 288.0605. ^1^H NMR (300 MHz, DMSO-d_6_) *δ*: 10.41 (s, 1H, -NH-), 8.71–8.69 (m, 1H, -ArH), 8.29–8.26 (m, 1H, -ArH), 7.63–7.58 (m, 2H, -ArH), 7.38–7.33 (m, 1H, -ArH), 7.17 (t, *J* = 8.9 Hz, 2H, -ArH), 4.26 (s, 2H, -CH_2_-) ppm.

##### 2-((4-Cyanopyridin-3-yl)thio)-N-(4-fluorophenyl)acetamide (LBJ-04)

The title compound was prepared from 3-chloro-4-cyanopyridine and **21b** following the synthesis route of **LBJ-01**. Yellow solid (1.2 g, 43%). m.p. 206–209 °C. HRMS *m/z* [M + H]^+^ calculated for C_14_H_11_FN_3_OS: 288.0601, found: 288.0603. ^1^H NMR (300 MHz, DMSO-d_6_) *δ*: 10.37 (s, 1H, -NH-), 8.69–8.67 (m, 1H, -ArH), 8.27–8.24 (m, 1H, -ArH), 7.61–7.56 (m, 2H, -ArH), 7.36–7.31 (m, 1H, -ArH), 7.18–7.12 (m, 2H, -ArH), 4.24 (s, 2H, -CH_2_-) ppm.

##### 2-((3-Cyanopyridin-4-yl)thio)-N-(4-fluorophenyl)acetamide (LBJ-05)

The title compound was prepared from 4-chloro-3-cyanopyridine and **21b** following the synthesis route of **LBJ-01**. White solid (1.3 g, 44%). m.p. 232–234 °C. HRMS *m/z* [M + H]^+^ calculated for C_14_H_11_FN_3_OS: 288.0601, found: 288.0603. ^1^H NMR (300 MHz, DMSO-d_6_) *δ*: 10.48 (s, 1H, -NH-), 8.86 (s, 1H, -ArH), 8.67 (d, *J* = 6.0 Hz, 1H, -ArH), 7.62–7.56 (m, 3H, -ArH), 7.21–7.13 (m, 2H, -ArH), 4.23 (s, 2H, -CH_2_-) ppm.

##### 2-((2-Cyanopyridin-3-yl)thio)-N-(4-fluorophenyl)acetamide (LBJ-06)

The title compound was prepared from 3-chloro-2-cyanopyridine and **21b** following the synthesis route of **LBJ-01**. Yellow solid (1.3 g, 45%). m.p. 211–213 °C. HRMS *m/z* [M + H]^+^ calculated for C_14_H_11_FN_3_OS: 288.0601, found: 288.0605. ^1^H NMR (300 MHz, DMSO-d_6_) *δ*: 9.63 (s, 1H, -NH-), 8.70–8.68 (m, 1H, -ArH), 8.46–8.43 (m, 1H, -ArH), 7.74–7.69 (m, 2H, -ArH), 7.57–7.53 (m, 1H, -ArH), 7.18 (t, *J* = 9.0 Hz, 2H, -ArH), 6.94 (s, 2H, -CH_2_-) ppm. 13C NMR (75 MHz, DMSO-d_6_) *δ*: 163.5, 158.0 (^1^*J*_CF_ = 240.0 Hz), 147.1, 146.8, 146.6, 135.1, 132.2, 131.7, 123.1 (^3^*J*_CF_ = 7.5 Hz), 122.5, 115.0 (^2^*J*_CF_ = 22.5 Hz), 42.8 ppm.

##### 2-((3-Cyano-6-methylpyridin-2-yl)thio)-N-(4-fluorophenyl)acetamide (LBJ-07)

The title compound was prepared from 2-chloro-3-cyanopyridine and **21b** following the synthesis route of **LBJ-01**. Yellow solid (1.4 g, 46%). m.p. 238–240 °C. HRMS *m/z* [M + H]^+^ calculated for C_15_H_13_FN_3_OS: 302.0758, found: 302.0758. ^1^H NMR (300 MHz, DMSO-d_6_) *δ*: 10.39 (s, 1H, -NH-), 8.11 (d, *J* = 9.0 Hz, 1H, -ArH), 7.61–7.57 (m, 2H, -ArH), 7.20–7.13 (m, 3H, -ArH), 4.17 (s, 2H, -CH_2_-), 2.5 (s, 3H, -CH_3_) ppm.

##### 2-((3-Cyano-6-(trifluoromethyl)pyridin-2-yl)thio)-N-(4-fluorophenyl)acetamide (LBJ-08)

The title compound was prepared from **18a** and **21b** following the synthesis route of **LBJ-01**. Yellow solid (1.5 g, 43%). m.p. 201–204 °C. HRMS *m/z* [M + H]^+^ calculated for C_15_H_10_F_4_N_3_OS: 356.0475, found: 356.0477. ^1^H NMR (300 MHz, DMSO-d_6_) *δ*: 10.42 (s, 1H, -NH-), 8.56 (d, *J* = 6.0 Hz, 1H, -ArH), 7.82 (d, *J* = 9.0 Hz, 1H, -ArH), 7.58–7.53 (m, 2H, -ArH), 7.15 (t, *J* = 9.0 Hz, 2H, -ArH), 4.24 (s, 2H, -CH_2_-) ppm. 13C NMR (75 MHz, DMSO-d_6_) *δ*: 164.0, 158.6 (^1^*J*_CF_ = 238.5 Hz), 149.0, 137.4, 135.4, 132.0, 128.0, 123.9, 122.9 (^3^*J*_CF_ = 7.5 Hz), 122.6, 114.9 (^2^*J*_CF_ = 22.5 Hz), 97.47, 30.65 ppm.

##### 2-((3-Cyano-6-(trifluoromethyl)pyridin-2-yl)thio)-N-(2-fluorophenyl)acetamide (LBJ-09)

The title compound was prepared from **18a** and **21d** following the synthesis route of **LBJ-01**. White solid (1.6 g, 45%). m.p. 208–211 °C. HRMS *m/z* [M + H]^+^ calculated for C_15_H_10_F_4_N_3_OS: 356.0475, found: 356.0473. ^1^H NMR (300 MHz, DMSO-d_6_) *δ*: 10.14 (s, 1H, -NH-), 8.56 (d, *J* = 9.0 Hz, 1H, -ArH), 7.83 (d, *J* = 9.0 Hz, 2H, -ArH), 7.30–7.23 (m, 1H, -ArH), 7.16–7.212 (m, 2H, -ArH), 4.33 (s, 2H, -CH_2_-) ppm.

##### N-(3-chloro-4-fluorophenyl)-2-((3-cyano-6-(trifluoromethyl)pyridin-2-yl)thio) acetamide (LBJ-10)

The title compound was prepared from **18a** and **21e** following the synthesis route of **LBJ-01**. White solid (1.9 g, 48%). m.p. 227–229 °C. HRMS *m/z* [M + H]^+^ calculated for C_15_H_9_ClF_4_N_3_OS: 390.0085, found: 390.0077. ^1^H NMR (300 MHz, DMSO-d_6_) *δ*: 10.58 (s, 1H, -NH-), 8.56 (d, *J* = 9.0 Hz, 1H, -ArH), 7.87–7.81 (m, 2H, -ArH), 7.46–7.35 (m, 2H, -ArH), 4.25 (s, 2H, -CH_2_-) ppm. 13C NMR (75 MHz, DMSO-d_6_) *δ*: 163.6, 158.4 (^1^*J*_CF_ = 238.5 Hz), 147.5, 145.5, 142.7, 137.5, 135.0, 133.5, 123.3 (^3^*J*_CF_ = 8.0 Hz), 116.4, 115.1 (^2^*J*_CF_ = 22.0 Hz), 101.5, 54.25 ppm.

##### 2-((3-Cyano-6-(trifluoromethyl)pyridin-2-yl)thio)-N-phenylacetamide (LBJ-11)

The title compound was prepared from **18a** and **21c** following the synthesis route of **LBJ-01**. Yellow solid (0.75 g, 23%). m.p. 230–233 °C. HRMS *m/z* [M + H]^+^ calculated for C_15_H_11_F_3_N_3_OS: 338.0569, found: 338.0579. ^1^H NMR (300 MHz, DMSO-d_6_) *δ*: 10.34 (s, 1H, -NH-), 8.55 (d, *J* = 9.0 Hz, 1H, -ArH), 7.81 (d, *J* = 9.0 Hz, 1H, -ArH), 7.54 (d, *J* = 6.0 Hz, 2H, -ArH), 7.30 (t, *J* = 9.0 Hz, 2H, -ArH), 7.05 (t, *J* = 7.5 Hz, 1H, -ArH), 4.25 (s, 2H, -CH_2_-) ppm. 13C NMR (75 MHz, DMSO-d_6_) *δ*: 164.90, 162.54, 144.32, 138.84, 128.65, 123.38, 119.24, 116.49, 116.45, 116.41, 114.54, 35.25 ppm.

##### 2-((3-Cyano-6-(trifluoromethyl)pyridin-2-yl)thio)-N-(p-tolyl)acetamide (LBJ-12)

The title compound was prepared from **18a** and **21f** following the synthesis route of **LBJ-01**. Yellow solid (2.30 g, 62%). m.p. 212–214 °C. HRMS *m/z* [M + H]^+^ calculated for C_16_H_13_F_3_N_3_OS: 352.0726, found: 352.0733. ^1^H NMR (300 MHz, DMSO-d_6_) *δ*: 10.24 (s, 1H, -NH-), 8.57–8.54 (m, 1H, -ArH), 7.81 (d, *J* = 9.0 Hz, 1H, -ArH), 7.41 (t, *J* = 4.5 Hz, 2H, -ArH), 7.10 (d, *J* = 6.0 Hz, 2H, -ArH), 4.24 (s, 2H, -CH_2_-), 2.25 (s, 3H, -CH_3_) ppm. 13C NMR (75 MHz, DMSO-d_6_) *δ*: 164.63, 162.57, 148.03, 144.29, 136.33, 132.31, 129.02, 119.26, 116.42, 116.38, 116.34, 114.53, 109.63, 102.88, 35.22, 20.38 ppm.

##### 2-((3-Cyano-6-(trifluoromethyl)pyridin-2-yl)thio)-N-(p-tolyl)acetamide (LBJ-13)

The title compound was prepared from **18a** and **21g** following the synthesis route of **LBJ-01**. Yellow solid (1.5 g, 42%). m.p. 217–219 °C. HRMS *m/z* [M + H]^+^ calculated for C_16_H_13_F_3_N_3_O_2_S: 368.0675, found: 368.0681. ^1^H NMR (300 MHz, DMSO-d_6_) *δ*: 10.20 (s, 1H, -NH-), 8.55 (d, *J* = 9.0 Hz, 1H, -ArH), 7.82 (d, *J* = 6.0 Hz, 1H, -ArH), 7.46–7.44 (m, 2H, -ArH), 6.89–6.86 (m, 2H, -ArH), 4.22 (s, 2H, -CH_2_-), 3.72 (s, 3H, -CH_3_) ppm. 13C NMR (75 MHz, DMSO-d_6_) *δ*: 164.38, 155.34, 144.29, 131.97, 120.83, 116.45, 116.38, 114.54, 113.81, 55.12, 54.78, 35.13, 27.31 ppm.

##### N-(3-Chloro-4-fluorophenyl)-2-((6-(trifluoromethyl)pyridin-2-yl)thio)acetamide (LBJ-14)

The title compound was prepared from 2-chloro-6-(trifluoromethyl)pyridine and **21e** following the synthesis route of **LBJ-01**. White solid (1.8 g, 52%). m.p. 86–88 °C. HRMS *m/z* [M-H]^–^ calculated for C_14_H_8_ClF_4_N_2_OS 363.0023, found 363.0017; ^1^H NMR (300 MHz, DMSO-d_6_) *δ*: 10.50 (s, 1H, -NH-), 7.95–7.86 (m, 2H, Ar-H), 7.70 (d, *J* = 9.0 Hz, 1H, Ar-H), 7.59 (d, *J* = 9.0 Hz, 1H, Ar-H), 7.45–7.37 (dd, *J* = 15.0 Hz, *J* = 9.0 Hz, 2H, Ar-H), 4.11 (s, 2H, -CH_2_-) ppm.

## Results and discussion

### Shape-based virtual screen

ROCS is a fast shape comparison application which developed based on the principle that molecules have similar shape if their volumes overlay well and any volume mismatch is a measure of dissimilarity^48^. Besides shape description ROCS includes a colour force field allowing for basic inclusion of chemical information in the screening process^49^^,^^50^. ROCS colour force field can be used to measure chemical features complementarity and refine shape-based superpositions based on chemical similarity. The colour force field is composed of SMARTS rules that determine chemical centres plus rules to determine how centres interact.

The combination of shape matching (shape Tanimoto) and functional group matching (colour Tanimoto) expressed as Tanimoto combo score. The 3D similarity was ranked by the Tanimoto ComboScore method. Both shape and colour score vary from 0 to 1 (besthits = 1), the combo score as the sum varies between 0 and 2^50^. In the chemical force field, a molecule is described by the spatial arrangement of six types: hydrogen-bond donors, hydrogen-bond acceptors, hydrophobes, anions, cations, and rings. Recently, several studies suggested that the bioactive conformation of a reference molecule is not needed for the enrichment of a shape-based screening^51^. These findings encouraged us to employ this complementary methodology (complementary to the shape of the target interaction site) as a screening model for the identification of novel scaffolds that possess similar structures with positive IDO1 inhibitors.

**Figure 3.**
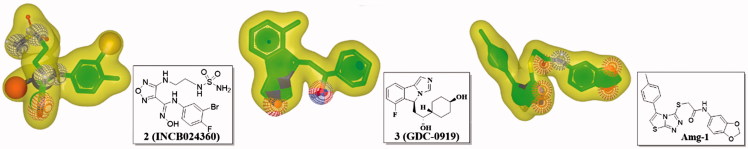
ROCS shape query derived from a low energy 3D conformation generated in MOE2016.09 of INCB024360 (2), GDC-0919 (3) and Amg-1. The green spheres illustrate ROCS ring features, the red spheres illustrate hydrogen-bond acceptors, and the blue spheres illustrate hydrogen-bond donors.

In this study, three representative compounds with diverse scaffolds, GDC-0919, INCB024360, and Amg-1, were selected as the reference molecule to generate the ROCS models. The 3D conformers of GDC-0919 and Amg-1 were obtained through the crystal structures (PDB code: 5EK4 and 4PK5, respectively). As for INCB024360, a low energy 3D conformer was generated using the MMFF94x forcefield (MOE 2016.08). The molecular shapes of these three compounds were depicted in yellow shadow (Figure 3). Then, the shaped queries of these three compounds were set to screen the Specs databased, containing approximately two hundred thousand molecules. First, over 180,000 compounds in Specs database were kept after removing PAINS (Pan Assay Interference Compounds structures) using the PAINS-remover online web server^52^. Then, the multiple conformations of different query molecules were calculated using OMEGA with a maximum of 300 conformations^53^. The top five hundred compounds for each query were carried forward for docking. There were some overlapped molecules obtained from these three queries, but we did not pick them out in this step.

### Docking results

#### Comparison of different crystal structures of IDO1-inhibitors

**Figure 4.**
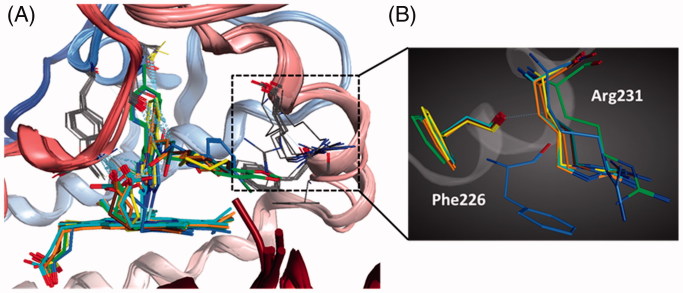
(A) The superimposition of six IDO1 crystal structures. (B) The significant movement of two representative residues (Phe226 and Arg231) in the catalytic pocket.

Up to date, nine crystal structures of IDO1 have been deposited in the RSCB Protein Data Bank (PDB). Among them, six structures with crystallographic resolutions higher than 3.0 Å were utilised in this work. These structures are all IDO1 with its potent inhibitors, including 4-phenylimidazole (4-PI, **8**, PDB code 2D0T)^36^, Amg-1 (**9**, PDB code 4PK5)^37^, **10** (PDB code 5EK2), **11** (PDB code 5EK3), **12** (PDB code 5EK4), and **13** (PDB code 5ETW)^38^. To compare the backbone conformation, 2D0T was used as the reference and the other structure was superposed onto it, and the RMSD was calculated (varies from 0.36 to 0.93 RMS deviation for the α-carbon atoms, Figure 4(A)). The low RMSD indicated that these IDO1 structures showed a high similarity. However, there is a significant movement of several residues, such as Phe226 and Arg231, in the catalytic pocket of IDO1-inhibitors, indicated that the pocket of IDO1 is flexible (Figure 4(B)).

#### Re-docking the crystal structure using different software

**Figure 5.**
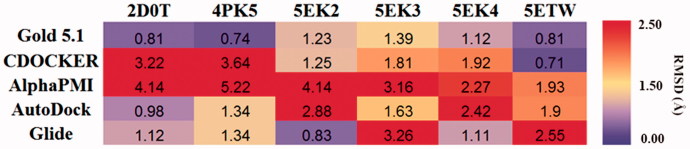
RMSD of ligands in cross-docking. The mean RMSD for the top-scoring pose of all the native ligands docked to the same protein are reported in the heat-map. The RMSD for each protein and docking protocol is represented by a colorimetric scale, going from purple to red from 0 to higher RMSD values.

In order to evaluate the accuracy of different docking programmes, including Gold 5.1 (The Cambridge Crystallographic Data Centre, UK), CDOCKET (BIOVIA, USA), Alpha PMI (Montreal, Canada), AutoDock (La Jolla, USA), and Glide (Los Angeles, USA), AutoDock, and Glide, we first performed the re-docking experiments. As shown in Table 1, among these docking software, GOLD5.1 software package with GoldScore scoring function possessed six high accuracy predictions (RMSD <1.0 Å). It indicated that Gold5.1 showed higher reproducibility than other software. For further validated the docking result, we carried the cross-docking experiment. Consistent with the result of native-docking, docking using the Gold5.1 had the smallest average RMSD (Figure 5). Hence, Gold5.1 was selected and employed to filter the molecular library for discovering the putative IDO1 inhibitors by VS. As for the selection of the IDO1 crystal structures, we noticed that the IDO1 pocket was very flexible and thus the rigid receptor docking will more than likely fail in finding the diverse scaffold against IDO1. Thus, we employed multiple crystal structures on constructing an ensemble docking to deal with protein flexibility. All of the six crystal structures were employed as docking template to identify structurally diverse IDO1 inhibitors.

**Table 1.** Information of all eight of the IDO1 PDB cognate ligands.

				Re-docking RMSD (Å)				
No.	PDB_ID	Resolution (Å)	PDB_Ligand	GOLD	CDOCKER	AlphaPMI	AutoDock	Glide
**1**	2D0T	2.3		0.70	1.11	0.92	0.82	0.65
**2**	2D0U	3.4	-CN	–	–	–	–	–
**3**	4PK5	2.79		0.32	0.43	0.57	0.72	1.01
**4**	4PK6	3.45		–	–	–	–	–
**5**	5EK2	2.68		0.48	1.08	1.03	0.64	0.44
**6**	5EK3	2.21		0.39	0.81	0.62	0.43	0.38
**7**	5EK4	2.64		0.48	0.78	0.35	0.42	0.56
**8**	5ETW	2.7		0.32	0.39	0.61	0.40	0.39

#### The results of molecular docking

**Figure 6.**
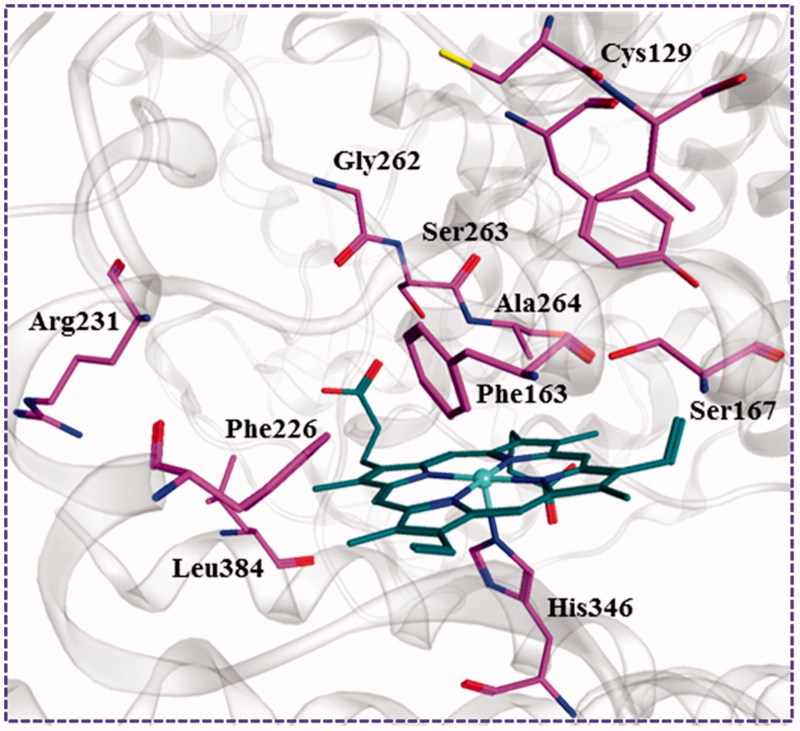
The key residues in binding site of IDO1. The binding pattern was generated form the cocrystal structure (PDB code: 4PK5) depicted using MOE 2016.09. The carbon atoms of haeme and the key residues in the active site of IDO1 were coloured in green and purple, respectively.

After the previous shape-based VS, the remaining compounds were submitted docking simulation using the genetic optimisation for ligand docking (Gold5.1) software package targeting multi-crystal IDO1 structures. The binding energy of the ligands with protein was predicted using GOLD score implemented in GOLD. The total GOLD score, which is represented as “Fitness,” was calculated from the contribution of hydrogen bonds and van der Waals interactions between protein and ligand as well as the contribution of intramolecular hydrogen bonds and intramolecular strain in the ligand. The binding site was defined as being any volume within 8 Å of the ligand in the crystal pose, and the residues Arg231, Leu384, Phe226, Gly262, Ser263, Ala264, Phe163, Ser167, Tyr126, Cys129, and His346 were found to be interacting residues as shown in Figure 6. All compounds were each docked to the six selected IDO1 structures, and the top 1% compounds were retained based on the Goldscore Fitness in each structure. Because this number included a large degree of similar compounds, with respect to scaffold and chemical class, a diversity analysis was undertaken using the program Diverse Subset (MOE 2016.09). Compounds with high GOLD fitness score usually could form hydrogen-bond interactions with Ala264, Ser167, and Ser263 and a coordinate bond with Fe^2+^ ion of haeme. In addition, as shown in the interaction between the native ligand and IDO1, the formed hydrophobic interactions with the hydrophobic resides Try126, Cys129, and Phe163 of IDO1 could improve the binding affinity. Top 10% of the molecules were recruited and further visually examined. In the process of visual inspection, we removed the reported compounds against IDO1 and ruled out the structures with the unreasonable binding modes discreetly (potential IDO1 inhibitors should possess functional group which could interact with the active site haeme of IDO1 and occupy the hydrophobic A pocket). Finally, 32 compounds were purchased form Specs for *in vitro* testing (Figure S1). Among these compounds **LVS-019** was discovered as the most potent IDO1 inhibitors.

#### Second round of screening based on compound LVS-019

As we knew, it is equivocal to determine the activity of a chemical scaffold judging by one compound due to the potential experimental error. In order to avoid the deviation in the subsequent molecular design and confirm the activity of screening hits, a further chemical similarity search based on **LVS-019** was performed. About 500 Compounds with similar molecular shape of **LVS-019** were screened out. The structural similarity of these molecules was then analysed using Functional-Class Fingerprints_6 (FCFP_6) molecular fingerprint^54^. Compounds with a Tanimoto coefficient over 0.5 were recognised as similar structures to **LVS-019**. After such screening, 48 derivatives of **LVS-019** were retained and processed to dock by Gold5.1 to rule out the structures with the unreasonable binding modes. Finally, 23 commercial available derivatives of **LVS-019** were retained and purchased to bioevaluation (Figure S2).

### Biological test

#### Inhibitory activities of screened compounds against purified recombinant IDO1 enzyme

**Figure 7.**
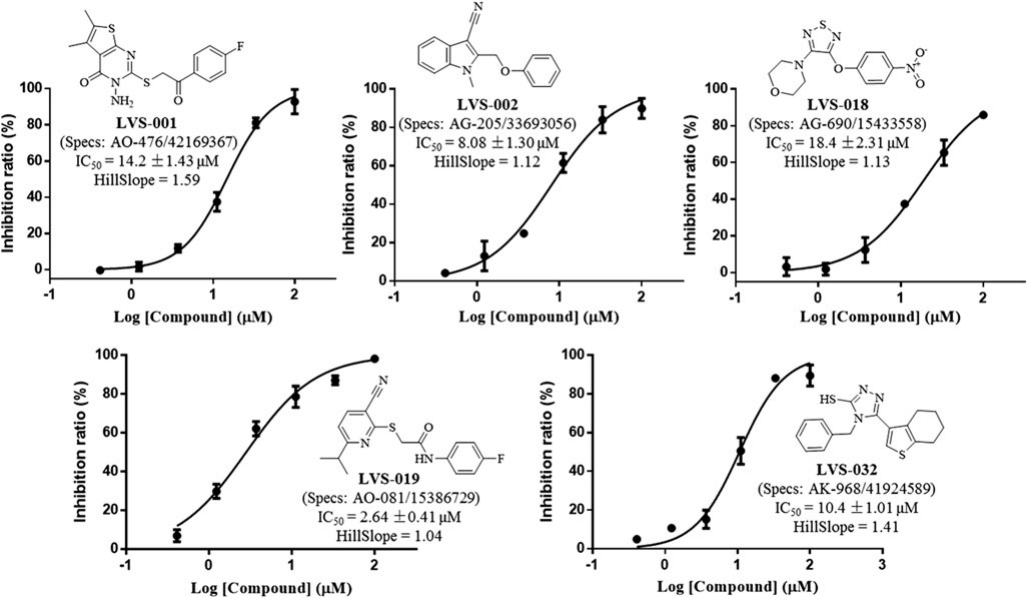
Five IDO1 inhibitors were found by VS from the database. The IDO1 inhibitory activity was obtained through enzymatic assay. The IC50 values were expressed as the mean of at least three independent determinations and are within ±15%.

**Figure 8.**
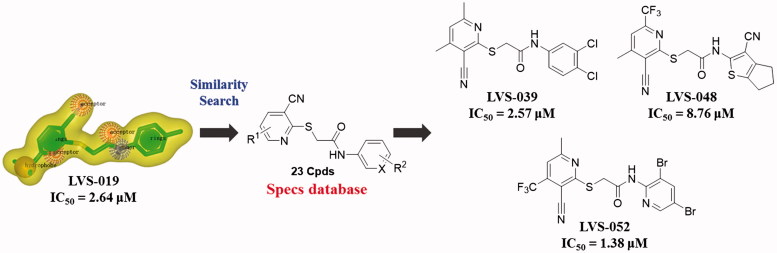
23 Derivatives were discovered based on the scaffold of LVS-019. Among these compounds, LVS-039, LVS-048, and LVS-052 showed similar potency compared with LVS-019. The IC50 values of other derivatives are shown in Table S2. The IC50 values were expressed as the mean of at least three independent determinations.

The potencies of the screened compounds in inhibiting the human IDO1 enzyme activity were initially evaluated by measuring the changes in absorbance values of the product produced from kynurenine and 4-dimethylaminobenzaldehyde at 480 nm^55^. To ensure that our compounds did not interfere with biological assays, representative compounds and water (Figure S3) with the addition of different concentrations of kynurenine in the absence of IDO1 protein. After *p*-dimethylaminobenzaldehyde (p-DMAB) was added, the kynurenine concentrations were determined by measuring the absorbance at OD 480 nm. The results showed that both of the test compounds did not interfere with the signal detection. Initial enzyme-based IDO1 inhibitory activity assay was conducted for 32 compounds obtained from VS at concentrations of 10 µM using L-1-MT and GDC-0919 analogue (racemic mixture of GDC-0919) as a positive control. The results are reported in Table S1. Based on the assay, five compounds (**LVS-001**, **LVS-002**, **LVS-018**, **LVS-019**, and **LVS-032**) revealed potential inhibitory activity to IDO1 with inhibition rates more than 50% at 10 µM. Then, we measured the dose response behaviours of these compounds, the IC_50_ values of these compounds are shown in Figure 7. Compound **LVS-019** with cyanopyridine scaffolds had the highest activity with IC_50_ value of 2.64 µM. Based on the promising scaffold of **LVS-019**, further 23 derivatives were discovered and evaluated. The structures and the IC_50_ values of these 23 derivatives are shown in Figures S2 and S4, and Table S2. Among these derivatives, several compounds (**LVS-039**, **LVS-048**, and **LVS-052**) showed similar potency compared with **LVS-019** (Figure 8). Comparing these cyanopyridine scaffold compounds with the control, L-1-MT and GDC-0919 analogue, had IC_50_ values of 42 µM and 0.81 µM, respectively. The results indicated that these compounds were more potent inhibitors than L-1-MT and was slightly weaker than GDC-0919 analogue. It should be mentioned that these hit molecules have not been optimised yet, and there existed a possibility for improving the binding affinity of each compound after careful molecular optimisation. Hence, the discovery of the new cyanopyridine scaffold derivatives with relatively good potency was encouraging.

#### Determination of IDO1 inhibitory activity in the HeLa cell-based method

**Figure 9.**
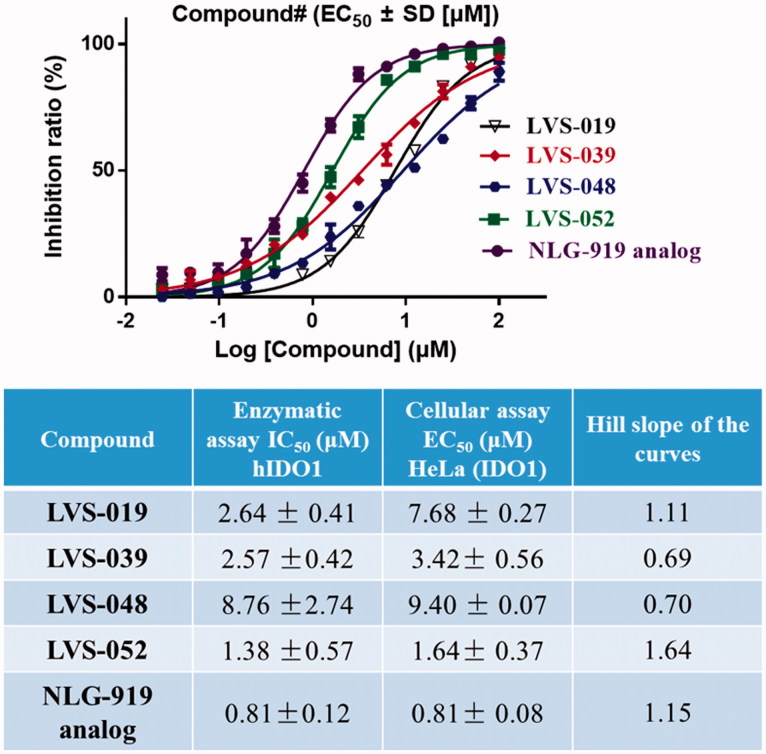
Dose-dependent inhibition of compounds LVS-019, LVS-039, LVS-048, LVS-052, and GDC-0919 analogue on cellular IDO1 inhibitory activity in the HeLa cell line. The table showed the enzymatic IC50, cellular IC50 and Hill slope of the curves of compounds LVS-019, LVS-039, LVS-048, LVS-052, and GDC-0919 analogue. The data were evaluated by Prism 6. The data are expressed as the mean ± SD calculated from three independent determinations.

The HeLa-based cellular assay is informative for drug development, as it evaluates not only the IDO1 inhibitory effect of the compounds but also their capacity to permeate the cell, their inhibition of tryptophan and kynurenine transport and their potential cytotoxicity^56^. The representative compounds (**LVS-019**, **LVS-039**, **LVS-048**, and **LVS-052**) were also assayed for their ability to inhibit tryptophan degradation and kynurenine production in the HeLa kynurenine assay. Figure 9 shows the dose response curves for IFN-γ stimulated HeLa cells (IDO1 high-expressed) incubated with **LVS-019**, **LVS-039**, **LVS-048**, and **LVS-052**. The Hill slope of these dose response curves did not deviate from 1, indicated that these compounds may be specific inhibitors of IDO1. These four compounds displayed cellular IDO1 inhibitory activity in the HeLa cell line with EC_50_ value in the range of 1.64–9.40 µM. More importantly, a good correlation between the recombinant enzyme-based and cellular-based assays confirmed that these cyanopyridine scaffold compounds can be developed to potent IDO1 inhibitors. In addition, since the inhibition of tryptophan degradation could simply be an effect of cytotoxicity, thus the determination of cell viability was necessary when reporting cellular IC_50_ values. In the initial work, all of the screened compounds were determined for their cell viability using the classical MTT assy. The results are shown in Figures S5 and S6, most of these compounds displayed negligible level of toxicity against HeLa cells at 50 µM.

### Predicted binding patters of potent inhibitors with cyanopyridine scaffold

**Figure 10.**
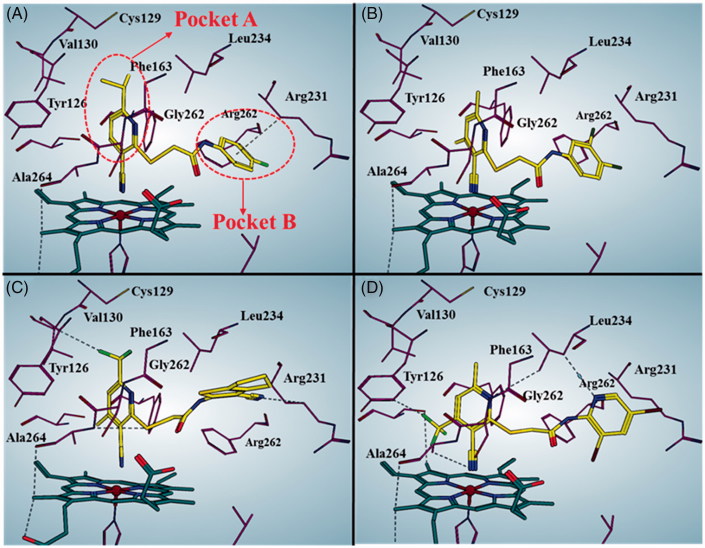
Predicted binding modes for compound LVS-019 (A), LVS-039 (B), LVS-048 (C), and LVS-052 (D) with IDO1. The pictures were generated using MOE 2016.09. The black dotted lines indicate interactions between ligand and receptor, including hydrogen bond, halogen bond, and H…π interactions.

Figure 10 shows the predicted binding mode of representative compounds **LVS-019**, **LVS-039**, **LVS-048**, and **LVS-052** to the top-scored protein structures (PDB code: 4PK5). The general molecular orientation and the spatial location of the chemical features of these inhibitors were similar to that of native ligand Amg-1. The nitrile group in pyrimidine ring of these compounds directly binds to the iron-haeme and pyrimidine ring is sitting at the pocket A. Furthermore, it is predicted that the ring B occupied the pocket B which is composed of residues Arg231, Phe262, and Leu234. More importantly, the ring B of these compounds could form π–π interaction with the Phe226. These four potent compounds were more effective than the other compounds, supported that the halogen or nitrile substitution at the B ring could form an additional electrostatic interaction of Arg231 which is necessary for improving the IDO1 inhibitory activity.

### Design, synthesis, and preliminarily evaluation of derivatives of LVS-019

Though these four screened compounds have been confirmed that possess potential IDO1 inhibitory activity and stable binding properties. However, there are still several defects for these compounds being promising lead compounds to further drug discovery, such as the moderate IDO1 inhibitory activity at micromole level and low water solubility (18 µg/mL). Thus, we designed several novel **LVS-019** derivatives (**LBJ-01∼LBJ-13**) with simplified structures and discussed the preliminary structure–activity relationship (SAR) of these series of compounds to find promising lead compound. In addition, to investigate the importance of the cyano group for IDO1 binding, we also designed and synthesised the compound **LBJ-14** which lacks the group.

**Scheme 1.**
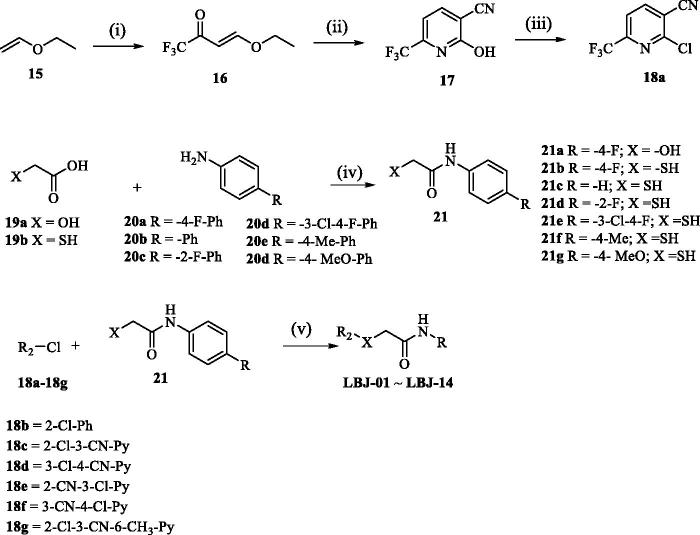
Reagents and conditions: (i) (CF_3_CO)_2_O, pyridine, CH_2_Cl_2_, 0 °C to 25 °C, 12 h, 60%; (ii) NCCH_2_CONH_2_, EtONa, EtOH, 0 °C to reflux, 12 h, 65%; (iii) POCl_3_, reflux, 2.5 h, 45%; (iv) 100–150 °C, 2–6 h, 84–89%; (v) K_2_CO_3_, DMF, 20 °C, 15–45 min, 33–48%. For the detailed R groups of compounds **LBJ-01–LBJ-14**, see Table 1.

The synthesis routes used to prepare derivatives **LBJ-01∼LBJ-13 are** shown in Scheme 1. The key intermediate **17** was synthesised starting from ethyl vinyl ether 15 in three steps by a modification of the previously report procedure^57^. Trifluoromethyl acetylation of **15** followed by condensation with cyanoacetamide provided pyridine **17**, which was subsequently converted to 2-chloropyridine by POCl_3_. Intermediate **21** was prepared through the neat acylation of substituted phenylamine (**20**) with glycolic acid (**19a**) or mercaptoacetic acid (**19b**). Finally, the target compounds (**LBJ-01∼LBJ-13**) were obtained by nucleophilic aromatic substitution using the intermediates **17a–17b** with **20** under weak base condition.

These 14 synthesised molecules were evaluated for their IDO1 inhibitory activity using an enzyme-based method. Encouragingly, compound **LBJ-08** (IC_50_ = 1.87 ± 0.83 µM) and **LBJ-10** (IC_50_ = 0.84 ± 0.05 µM) showed improved IDO1 inhibitory activity than **LVS-019** (IC_50_ = 2.64 ± 0.94 µM) (Table 2, Figure S7). The SAR studies demonstrated that the remove of the isopropyl group (**LBJ-03**) would reduce IDO1 inhibitory activity. **LBJ-1** with oxygen atom at the linker showed worse IDO1 activity compared with **LBJ-3**, indicated that the sulphur atom at the linker also has positive effect for the IDO1 inhibitory activity. Changing the 3-cyanopyridine scaffold (**LBJ-03**) to benzene (**LBJ-02**) or other substituted pyridine with nitrile group at different positions (**LBJ-04∼LBJ-06**) failed to show stronger inhibition efficiency. The 5-trifluoromethyl substituted cyanopyridine compound (**LBJ-08**) showed stronger IDO1 inhibitory activity than the unsubstituted compound **LBJ-03**. Based on **LBJ-08**, further modification focussed on the B ring, a series of different anilines substituted compounds (**LBJ-09∼LBJ-13**) was synthesised to explore the SAR, and most of the assayed compounds were less potent than **LBJ-08** except for **LBJ-10** which showed improved potency to that of **LBJ-08**. Compound **LBJ-14** was much weaker than compound **LBJ-10**, indicated the necessity of the presence of cyano group in these series of compounds. With IDO1 inhibitory activity at the nanomole level, compound **LBJ-10** could be a promising IDO1 inhibitor for further study. We also determined the solubility of these compounds using the “goldstandard” Avdeef–Bucher potentiometric titration method. To our disappointment, except for compound **LBJ-03** with four times more soluble (75 µg/mL) than **LVS-019**, other compounds were on the same level with it. Thus, we would further focus on improving the drug-like properties of these series of compounds in the near future.

**Table 2.** Inhibitory activity of the cyanopyridine scaffold derivatives against purified human IDO1 enzyme.

Compound	Structure	Enzymatic assay (IC_50_, μM)^a^	Compound	Structure	Enzymatic assay (IC_50_, μM)^a^
LBJ-01		19.5 ± 0.28	LBJ-09		5.81 ± 1.20
LBJ-02		23.1 ± 0.67	LBJ-10		0.84 ± 0.33
LBJ-03		9.14 ± 0.38	LBJ-11		9.61 ± 0.71
LBJ-04		43.6 ± 0.85	LBJ-12		17.9 ± 1.28
LBJ-05		18.0 ± 0.13	LBJ-13		23.6 ± 2.11
LBJ-06		9.76 ± 0.32	LBJ-14		35.7 ± 1.25
LBJ-07		20.6 ± 1.12	LVS-019		2.64 ± 0.94
LBJ-08		1.87 ± 0.83	GDC-0919 analogue	–	0.81 ± 0.04

^a^The IC_50_ values were the means of more than three independent experiment sets.

### Spectroscopic and reversibility analysis of LBJ-10 binding

**Figure 11.**
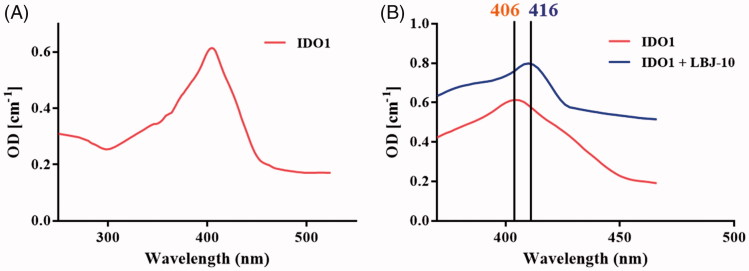
Spectroscopic analysis of LBJ-10 binding. UV spectra of ferric IDO1 without (A) and with 20 μΜ concentration of compound LBJ-10 (B). The Soret’s ratio (A404/A280) of purified IDO1 was 2.15.

A UV–visible spectra study was performed to evaluate the binding of the representative compound **LBJ-10** (Figure 11). The absorption spectrum of the haeme group is highly sensitive to the changes in the polarity of haeme surrounding upon the ligand or substrate binding, leading to the change of spectra properties of the haeme^38^. The UV–visible spectra of ferric IDO1 was measured in the presence and absence of compound **LBJ-10**. In the absence of compound **LBJ-10**, the absorption spectrum of ferric IDO1 exhibited a Soret peak at 406 nm. When in the presence of compound **LBJ-10**, the spectra shifted to 416 nm which demonstrated that compound **LBJ-10** binds to ID O1 and coordinates with the iron of the haeme group.

**Figure 12.**
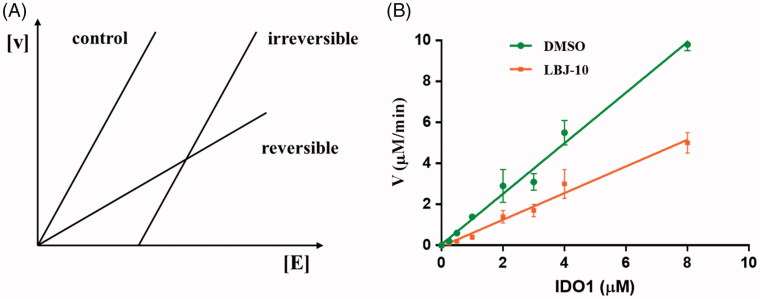
Inhibition type of LBJ-10. (A) Determination of the inhibition type. Reaction rate [V] is plotted against enzyme amount [E]. Curves with different slopes represent reversible and irreversible inhibitions, respectively. (B) Inhibition types of LBJ-10. Plot of reaction rate [V] against enzyme amount [E]. The concentrations of L-tryptophan and the inhibitor were 50 and 1 μM, respectively.

We also carried the additional experiment to determine the reversibility of compound **LBJ-10**. Detailed kinetics analysis was performed on **LBJ-10** based on plotting a function of the rate (V) against the enzyme amount ([E]) (Figure 12(A))^58^^,^^59^. The result showed that the representative **LBJ-10** was categorised as reversible inhibitor (Figure 12(B)). More specific kinetic and crystal structure experiments would be carried to verify the inhibitory mode and binding mode of these series of compounds.

## Conclusions

With the combined strategy of similarity search and molecular docking study, a diverse range of scaffolds of IDO1 inhibitors were identified from the Specs database. The shape-based screening model was developed using the ROCS method, based on the reported representative IDO1 inhibitors INCB024360, GDC-0919, and Amg-1. Subsequently, optimal docking process was further employed to screen the compounds and 32 hit compounds were finally discovered. Testing the screened hit compounds has led to several submicromolar inhibitors with novel scaffolds. Especially for compounds **LVS-019** with cyanopyridine scaffold, showed the best IDO1 inhibitory activity. To discover more compounds with similar structures to **LVS-019**, a ROCS model was then generated on the basis of **LVS-019** and then the second-round VS was carried out. Then the hits obtained by similarity search were further evaluated by GOLD, and 23 compounds were purchased to test *in vitro* leading to the discovery of another three potent compounds (**LVS-039**, **LVS-048**, and **LVS-052**). To improve potency of these hit compounds, we designed and synthesised another 14 derivatives of **LVS-019** through rational drug designation. As we expected, compound **LBJ-10** showed improvement potency compared to the hit compound and displayed comparable potency to the control GDC-0919 analogue. In this direction, the *in silico* approach described can also be used to screen existing database to identify derivatives with desired activity. Further studies on the molecular optimisation of the other hits to provide some other potent IDO1 inhibitors with novel scaffolds are currently underway.

## Supplementary Material

supporting_Information.doc
