# Supplementary material for: Discovery of cyanopyridine scaffold as novel indoleamine-2,3-dioxygenase 1 (IDO1) inhibitors through virtual screening and preliminary hit optimisation
Source: J Enzyme Inhib Med Chem. 2019 Jan 2;34(1):250–63. doi: 10.1080/14756366.2018.1480614 (PMC6327983; doi:10.1080/14756366.2018.1480614)
Supplement: supporting_Information.doc [file IENZ_A_1480614_SM5119.doc]

## Supporting Information

**Discovery of** **cyanopyridine scaffolds as novel indoleamine-2,3-dioxygenase 1 (IDO1) inhibitors through a multistep structure-based virtual screening**

Xi Xu^a,b^, Jie Ren^a,b^, Yinghe Ma^a,b^, Hongting Liu^a^, Quanjin Rong^a^, Yifan Feng^a^, Yameng Wang^a^, Yu Cheng^a^, Ruijia Ge^c^, Zhiyu Li ^a,b*^, and Jinlei Bian^a,b*^

^a^ State Key Laboratory of Natural Medicines, Jiangsu Key Laboratory of Drug Design and Optimization, China Pharmaceutical University, Nanjing 210009, China

^b^ Department of Medicinal Chemistry, School of Pharmacy, China Pharmaceutical University, Nanjing 210009, China

^c^The Madeira School,8328 Georgetown Pike Mclean, Virginia 221022

**Corresponding authors. E-mail: zhiyuli@cpu.edu.cn (Z. Li), bianjl@cpu.edu.cn (J. Bian)*

*Corresponding authors

E-mail addresses:

bianjl@cpu.edu.cn (J.L. Bian)

zhiyuli@cpu.edu.cn (Z.Y. Li)

**Fig. S1** The structure of the 32 screened compounds through the first-round combined virtual screen method.

**Table S1.** The inhibition ratio of 32 screened compounds against IDO1 @10 μM.

| **No.** | **Database ID** | **Inhibition ratio (%) @10 μM***^a^* | **No.** | **Database ID** | **Inhibition ratio (%) @10 μM***^a^* |
| --- | --- | --- | --- | --- | --- |
| **LVS-001** | AO-476/42169367 | 54.33 ± 0.06 | **LVS-018** | AG-690/15433558 | 51.32 ± 0.38 |
| **LVS-002** | AG-205/33693056 | 63.33 ± 1.02 | **LVS-019** | AO-081/15386729 | 83.65 ± 0.92 |
| **LVS-003** | AG-205/05238057 | NA | **LVS-020** | AG-205/13051083 | 0.11 ± 1.98 |
| **LVS-004** | AH-262/36680042 | 9.65 ± 0.09 | **LVS-021** | AH-487/42353530 | NA |
| **LVS-005** | AQ-750/41790223 | 30.73 ± 0.93 | **LVS-022** | AP-853/42938598 | 21.29 ± 0.22 |
| **LVS-006** | AG-205/08032009 | 15.83 ± 0.18 | **LVS-023** | AE-406/41057009 | 10.22 ± 1.11 |
| **LVS-007** | AG-690/36532062 | NA | **LVS-024** | AI-204/31682004 | NA |
| **LVS-008** | AG-205/36710015 | 4.31 ± 0.04 | **LVS-025** | AT-051/43421447 | NA |
| **LVS-009** | AG-690/34448040 | NA | **LVS-026** | AO-476/43250104 | NA |
| **LVS-010** | AG-690/36104037 | NA | **LVS-027** | AK-968/40945597 | 3.37 ± 0.04 |
| **LVS-011** | AG-205/10457052 | NA | **LVS-028** | AK-968/15605124 | NA |
| **LVS-012** | AO-365/43401709 | NA | **LVS-029** | AK-778/07165027 | 9.91 ± 0.65 |
| **LVS-013** | AG-205/40775917 | 14.15 ± 0.38 | **LVS-030** | AP-853/43405429 | 2.00 ± 0.28 |
| **LVS-014** | AH-487/41956097 | 11.62 ± 1.19 | **LVS-031** | AF-399/41945572 | NA |
| **LVS-015** | AO-081/41965504 | 11.05 ± 0.02 | **LVS-032** | AK-968/41924589 | 61.83 ± 1.02 |
| **LVS-016** | AN-988/42110136 | NA | **NLG-919** |  | 85.26 ± 0.03 |
| **LVS-017** | AG-667/11910001 | NA |  |  |  |

*^a^*Values are expressed as the mean of at least three independent determinations. NA = no activity

**Fig. S2**. The structures of the 23 derivatives of **LVS-19** discovered through shape-based screen.

**Table S2**. The IC_50_ values against IDO1 enzyme of 23 screened derivatives of **LVS-019**.

| **No.** | **Database ID** | **IC_50_ (μM)*^a^*** | **No.** | **Database ID** | | **IC_50_ (μM)*^a^*** |
| --- | --- | --- | --- | --- | --- | --- |
| **LVS-033** | AK-777/37037040 | 36.60 ± 5.46 | **LVS-045** | AM-807/14147255 | | 26.03 ± 9.64 |
| **LVS-034** | AN-919/15231402 | 66.19 ± 14.28 | **LVS-046** | AM-807/14147258 | | Not Converged |
| **LVS-035** | AN-988/14770027 | 27.63 ± 6.82 | **LVS-047** | AM-807/14147267 | | 21.23 ± 7.10 |
| **LVS-036** | AG-690/40697570 | 78.71 ± 8.46 | **LVS-048** | AM-807/14147297 | | 8.76 ± 1.74 |
| **LVS-037** | AK-777/12226045 | 54.67 ± 4.70 | **LVS-049** | AM-807/14147288 | | 14.76 ± 3.14 |
| **LVS-038** | AK-968/13026008 | Not Converged | **LVS-050** | AM-807/14147303 | | Not Converged |
| **LVS-039** | AK-968/13026017 | 2.57 ± 0.42 | **LVS-051** | AM-807/14147707 | | Not Converged |
| **LVS-040** | AM-807/13616371 | 74.67 ± 15.23 | **LVS-052** | AG-205/33674001 | | 1.38 ± 0.57 |
| **LVS-041** | AM-807/13616531 | 55.08 ± 10.07 | **LVS-053** | AM-807/42860087 | | Not Converged |
| **LVS-042** | AM-807/13616533 | 38.49 ± 6.09 | **LVS-054** | AN-848/40160475 | | Not Converged |
| **LVS-043** | AG-690/13770047 | 21.72 ± 3.18 | **LVS-055** | AE-641/00167027 | | Not Converged |
| **LVS-044** | AM-807/14146438 | 49.30 ± 10.02 | **GDC-0919 analog** | |  | 0.81 ± 0.12 |
|  |  |  | **L-1-MT** | |  | 42.11 ± 8.05 |

*^a^*Values are expressed as the mean of three independent determinations.

**Fig S3**. Spectrophotometry analysis of compound **LVS-019** and **LBJ-10** at 480 nm without IDO1 protein in different concentrations kynurenine. After p-dimethylaminobenzaldehyde (p-DMBA) (100 μL, 2% w/v in acetic acid) was added, the kynurenine concentrations were determined by measuring the absorbance at OD 480 nm. Both of the compounds show no interference in the signal of biological assay.


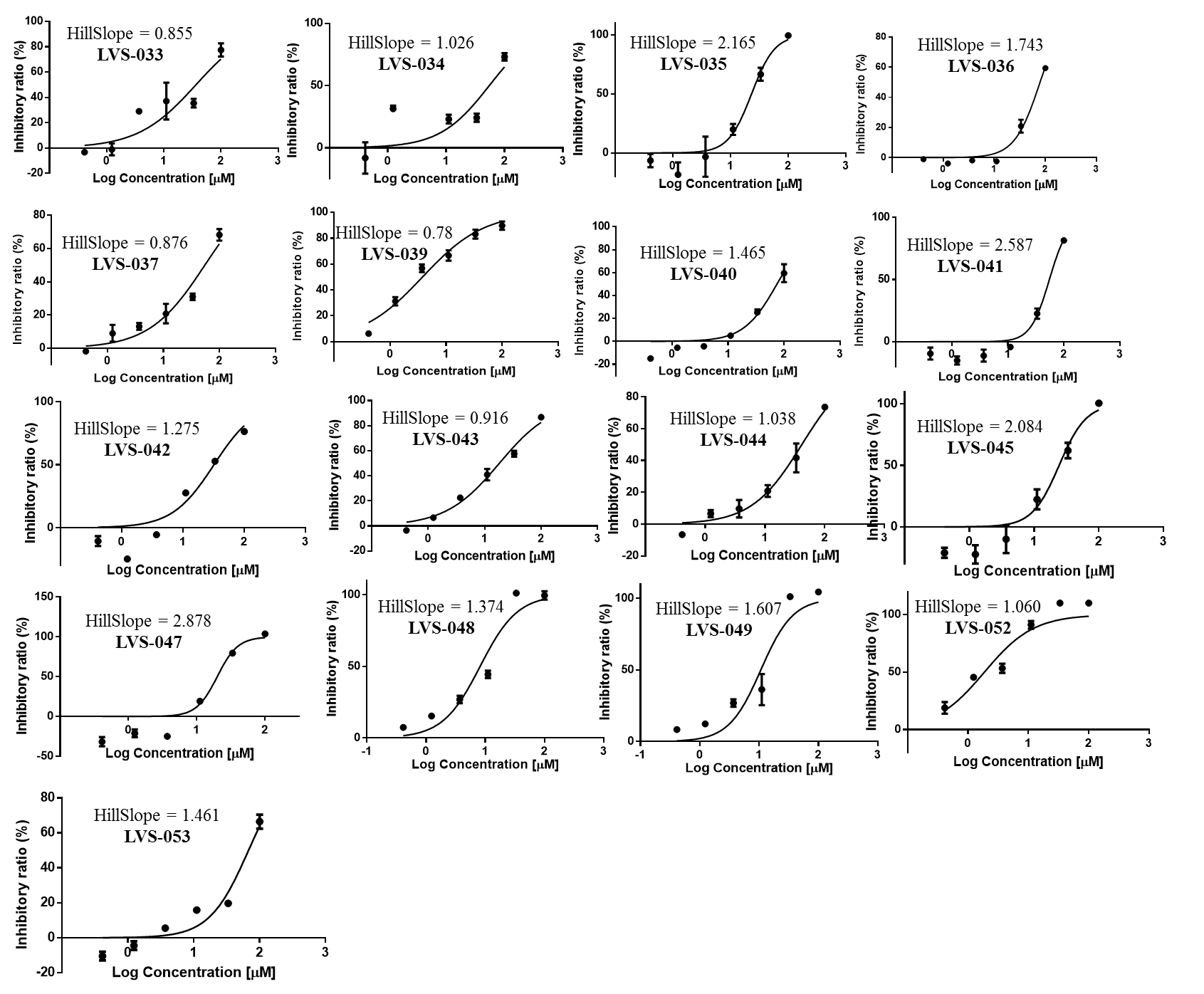


**Fig. S4**. The enzymatic IC_50_ curves for all compounds together with their HillSlope coefficients.


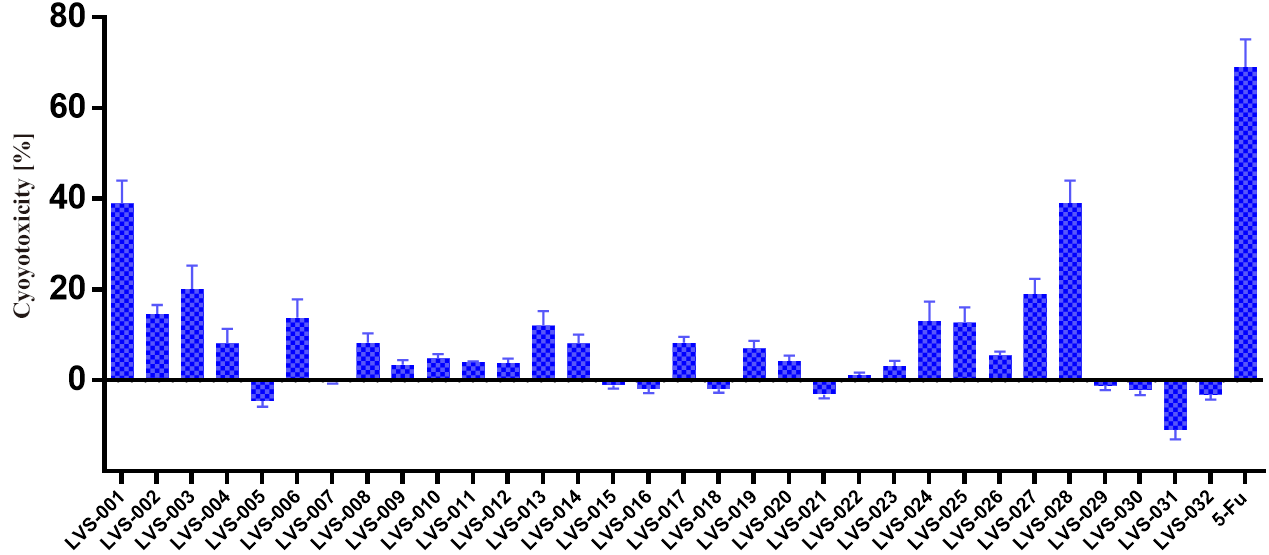


**Fig. S5**. The toxicity test of 32 screened compounds using MTT method. The inhibition ratio of these compounds @50 μM. Values are expressed as the mean of three independent determinations. 5-Fu represents 5-fluorouracil.


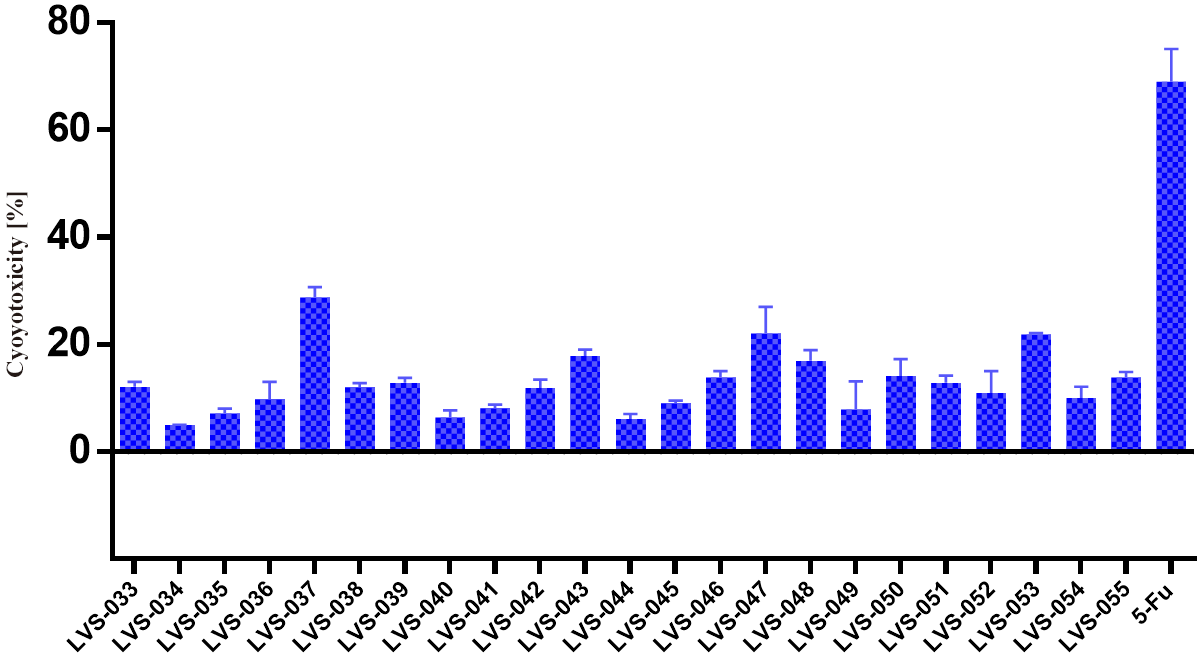


**Fig. S6**. The toxicity test of 23 derivatives of **LVS-19** using MTT method. The inhibition ratio of these compounds @50 μM. Values are expressed as the mean of three independent determinations. 5-Fu represents 5-fluorouracil.


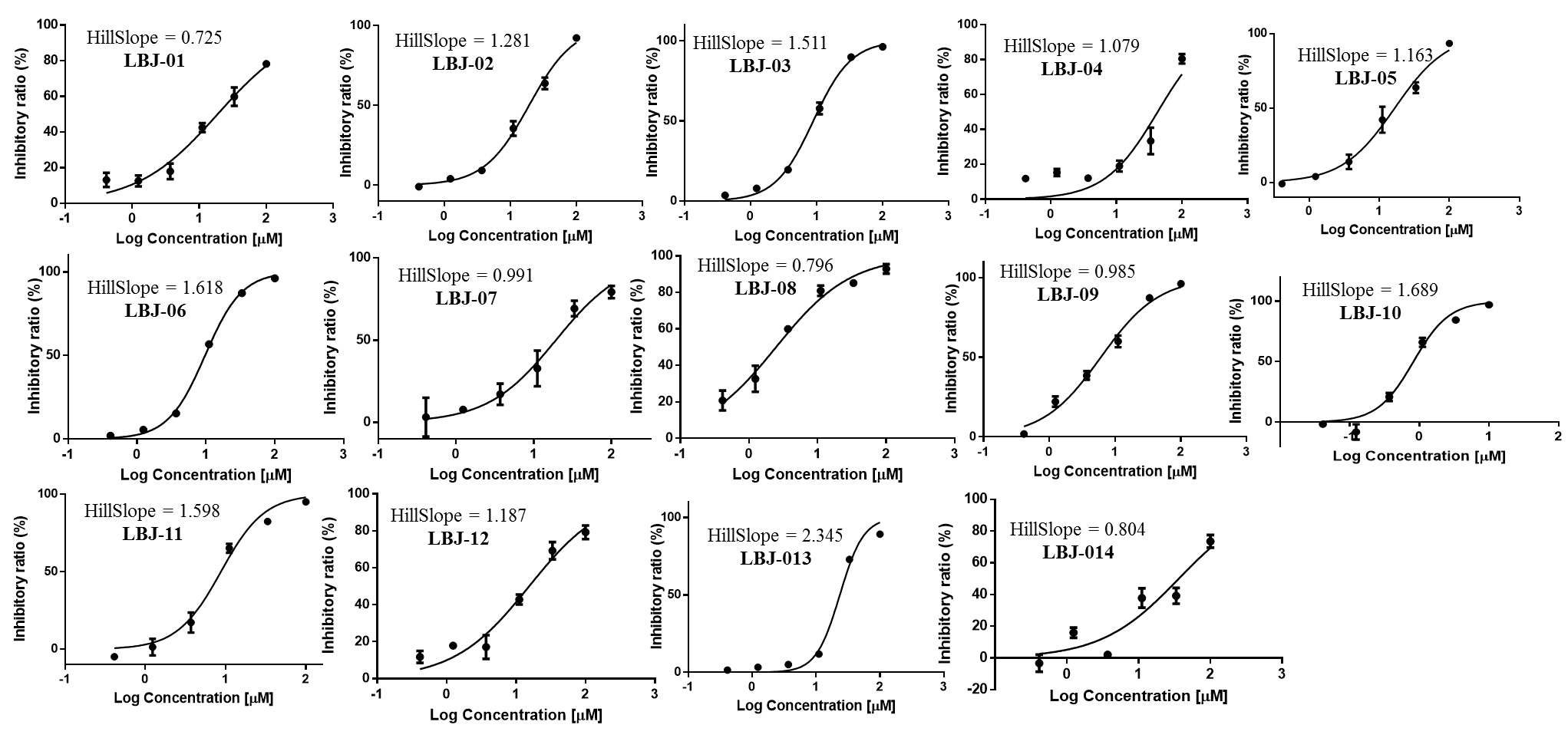


**Fig. S7**. The enzymatic IC_50_ curves for all compounds together with their HillSlope coefficients (**LBJ-001~LBJ-014**).

**Table S3**. The recent available PDB_ID human IDO1-inhibitors

| **No.** | **PDB_ID** | **Resolution (Å)** | **PDB_Ligand** |
| --- | --- | --- | --- |
|  |  |  |  |
| **1** | 5XE1 | 3.2 |  |
| **2** | 6AZU | 2.82 | - |
| **3** | 6AZV | 2.76 |  |
| **4** | 6AZW | 2.78 |  |
| **5** | 5WNU | 2.4 |  |
| **6** | 5WMV | 2.6 |  |
| **7** | 5WMW | 3.03 |  |
| **8** | 5WMX | 2.69 |  |
| **9** | 5WN8 | 2.5 |  |
| **10** | 6F0A | 2.26 |  |
| **11** | 5WHR | 2.28 |  |
| **12** | 4U72 | 2.0 | - |
| **13** | 4U74 | 2.31 | - |

**NMR spectra**


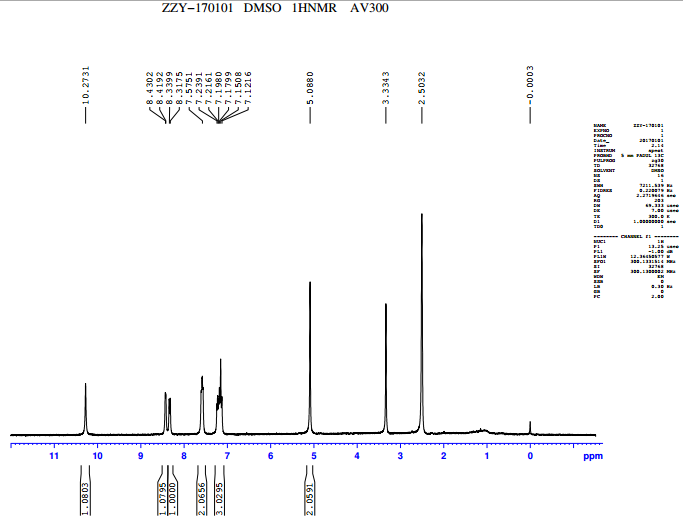


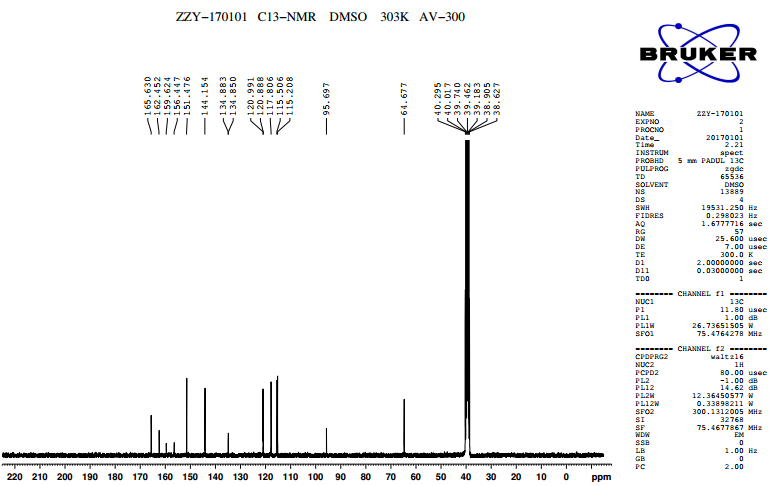


**LBJ-01**


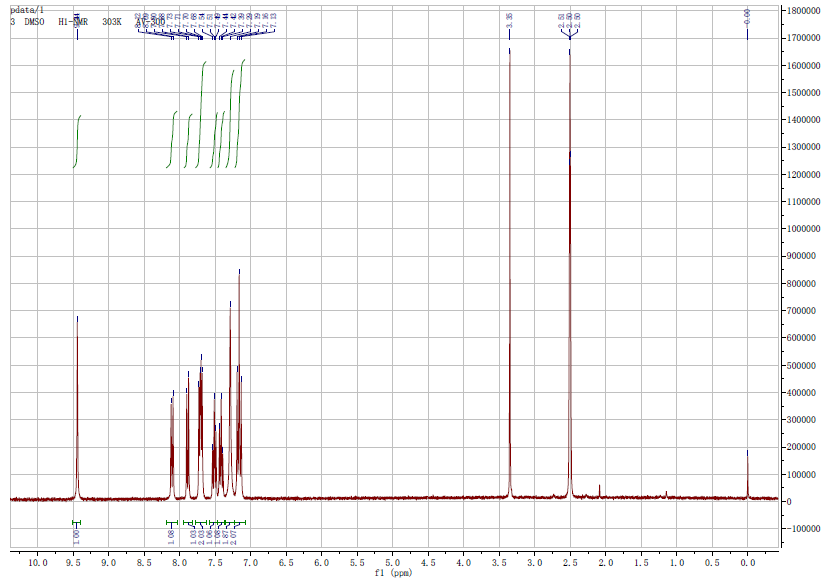


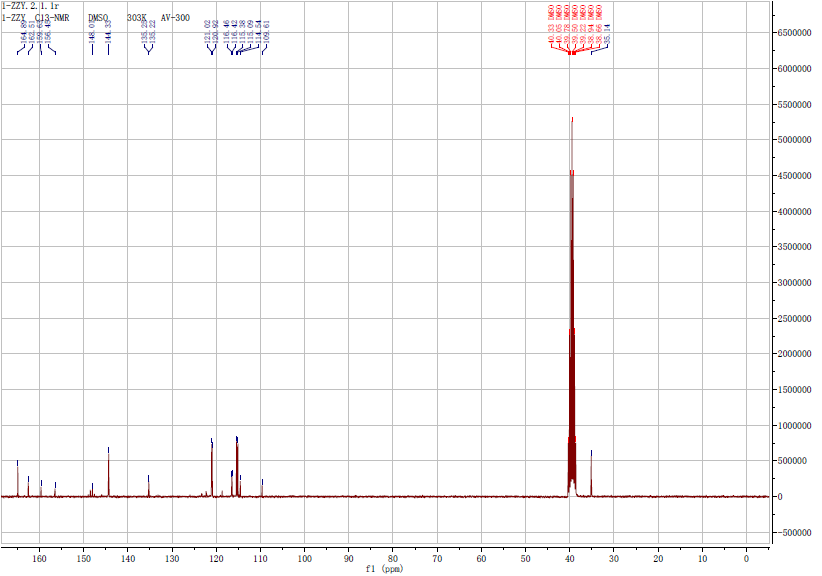


**LBJ-02**


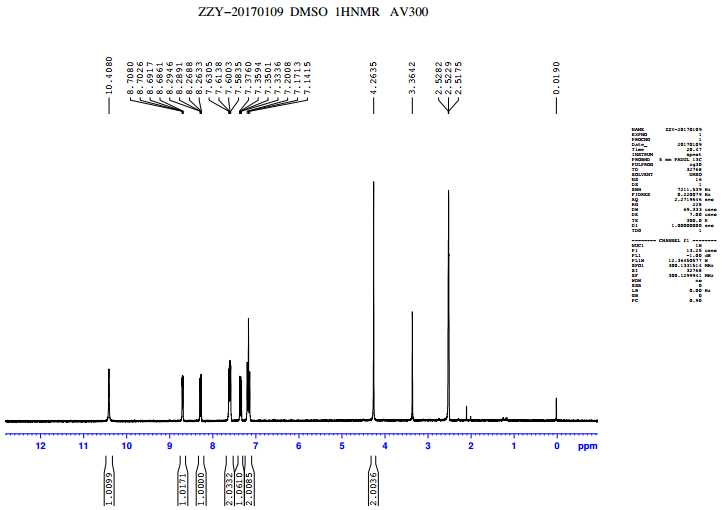


**LBJ-03**


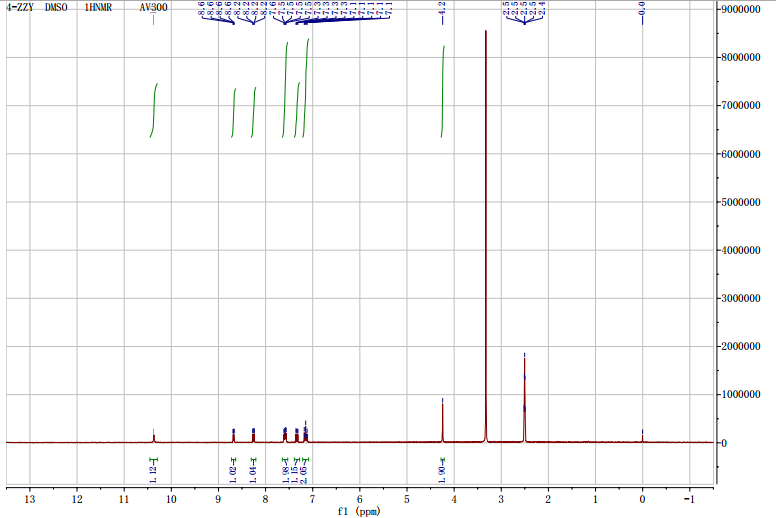


**LBJ-04**


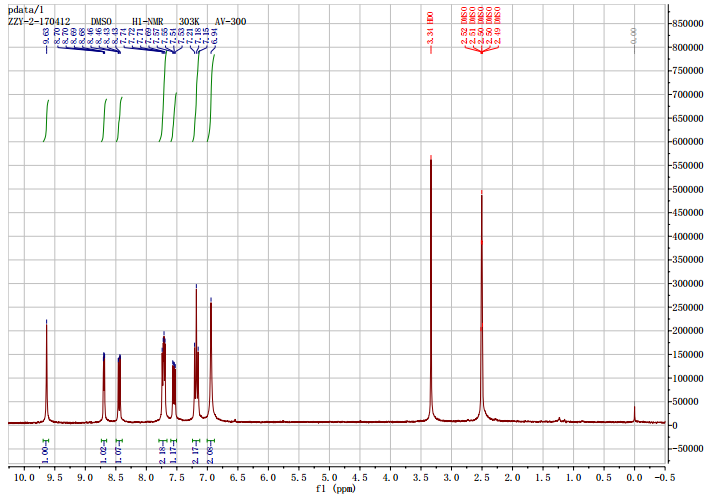


**LBJ-05**


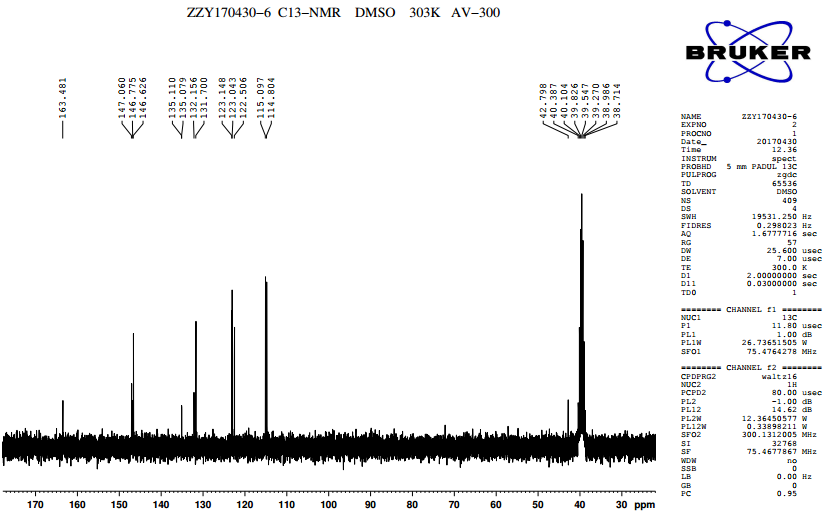


**LBJ-06**


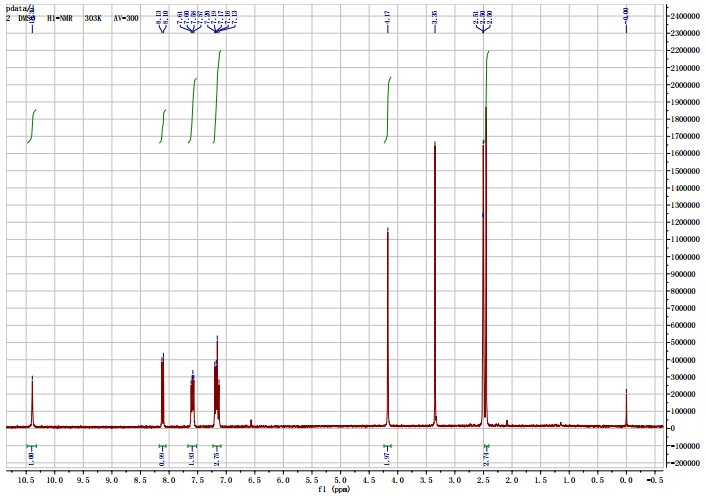


**LBJ-07**


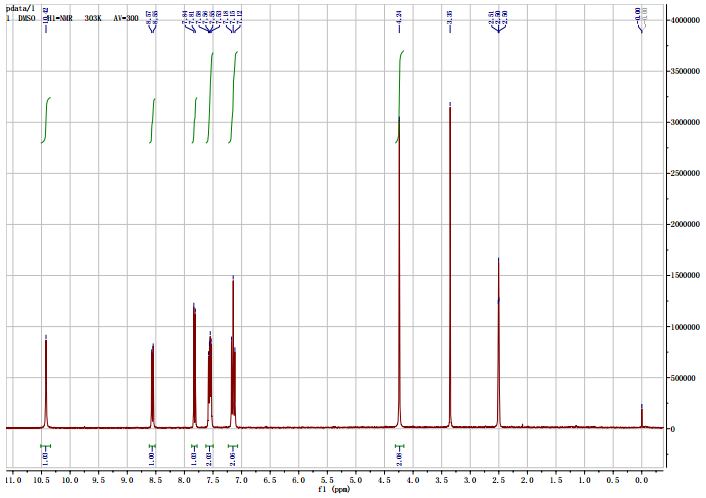


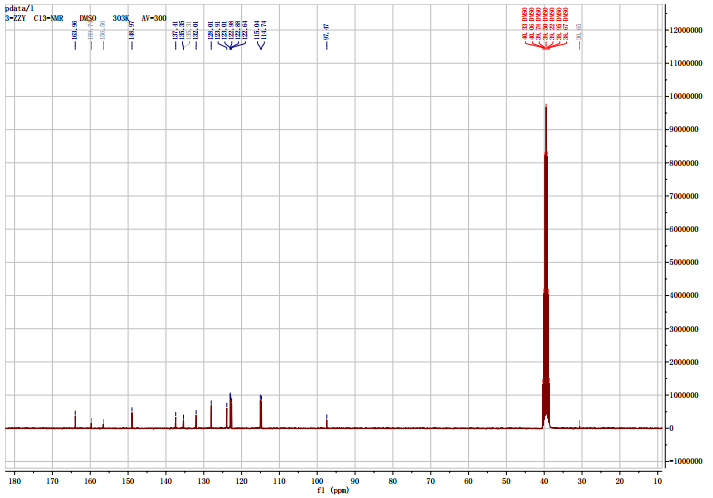


**LBJ-08**


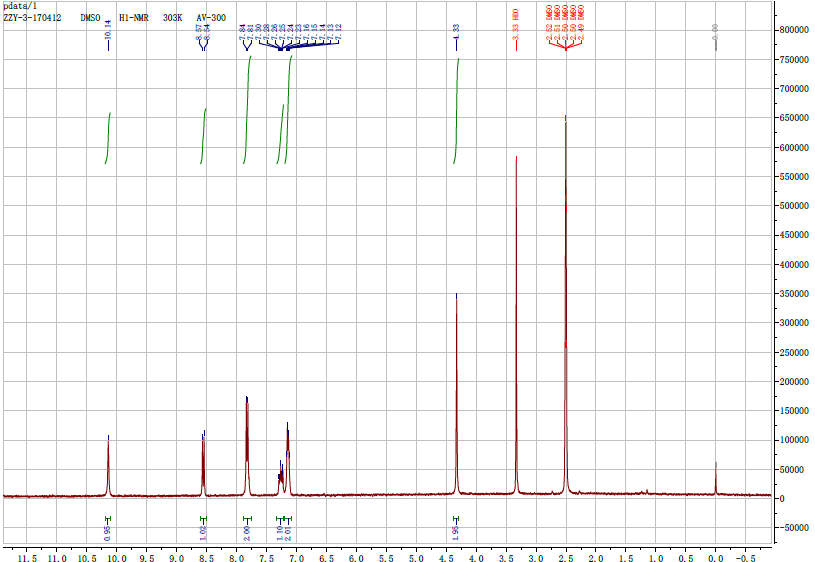


**LBJ-09**


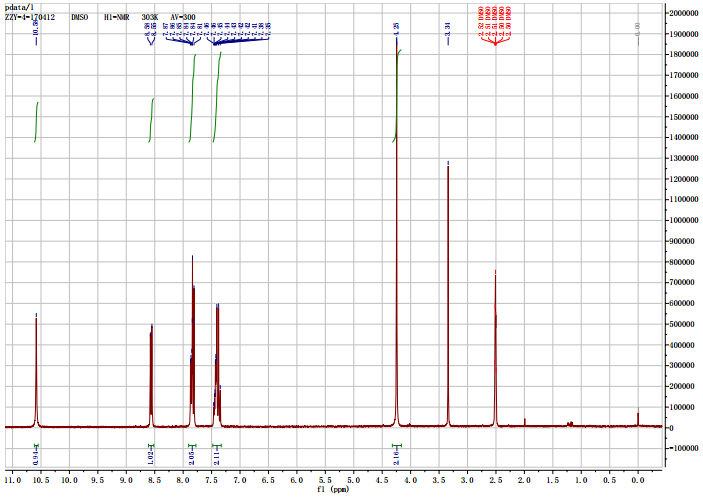


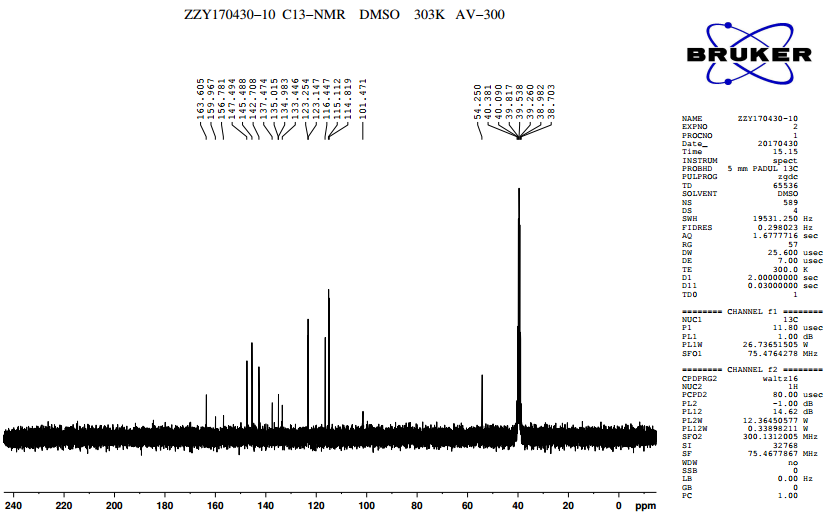


**LBJ-10**


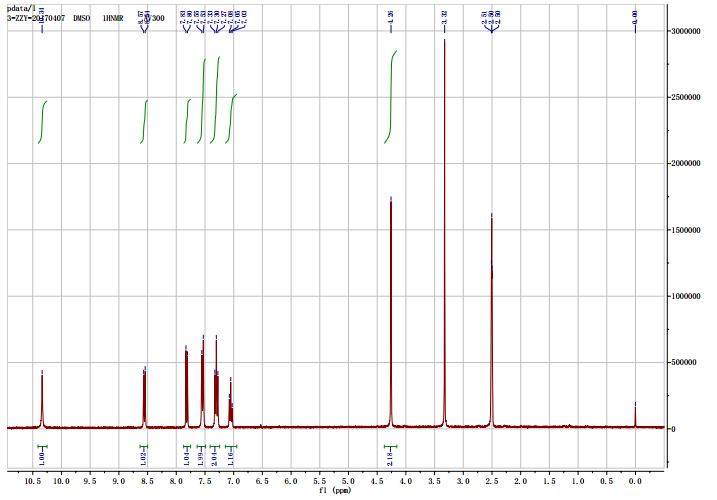


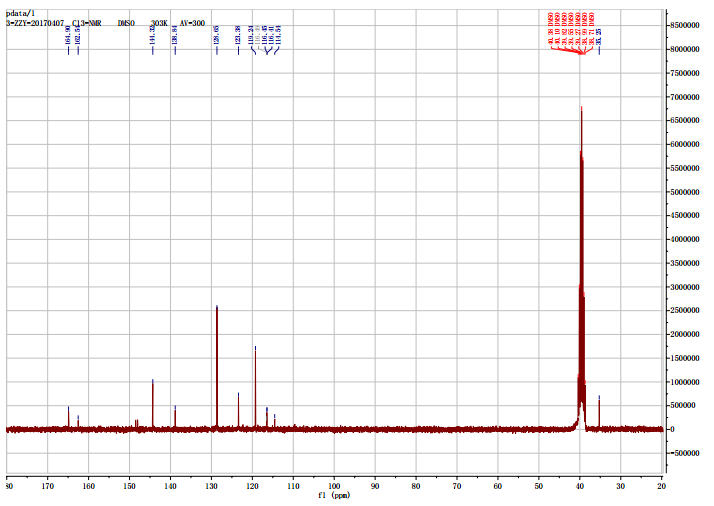


**LBJ-11**


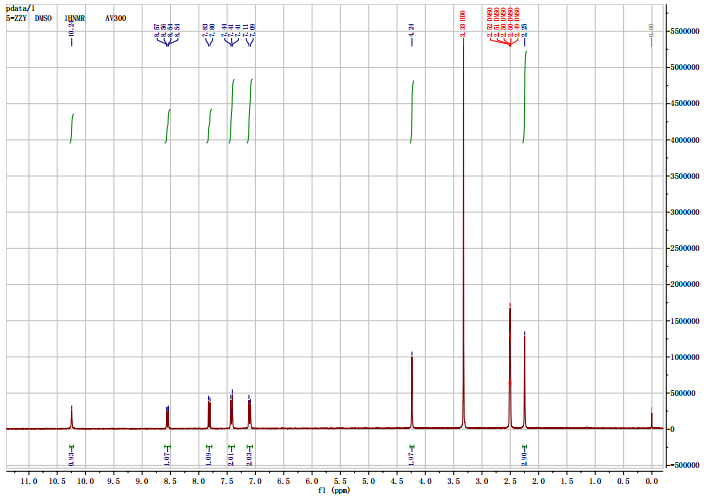


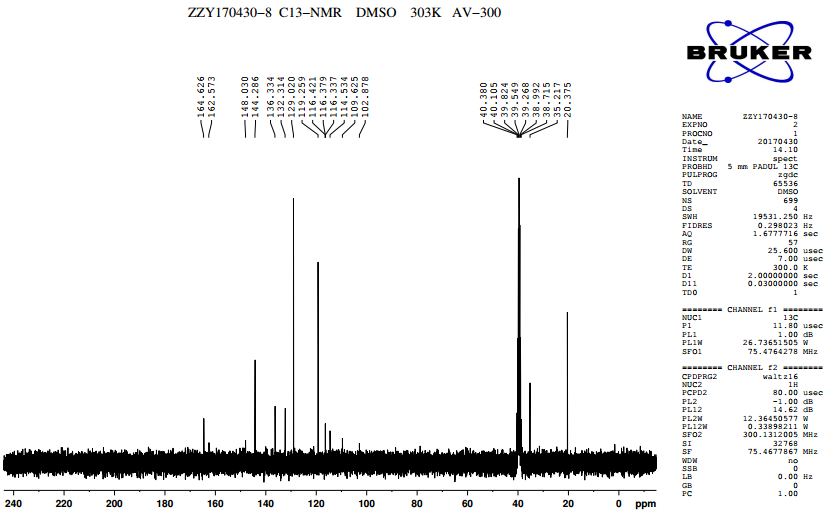


**LBJ-12**


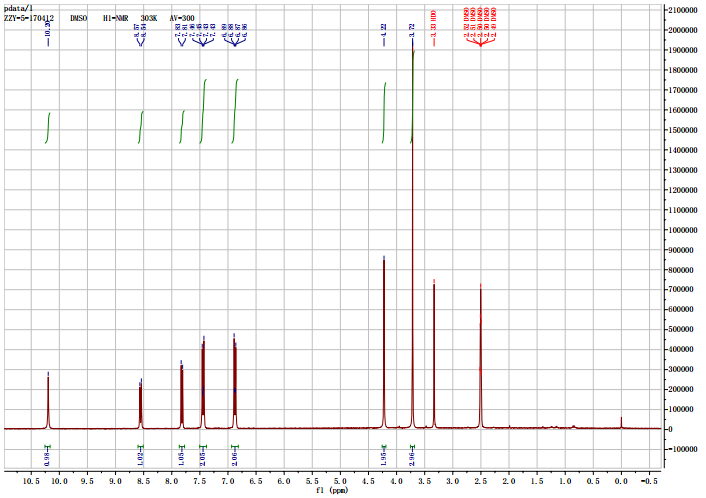


**LBJ-13**


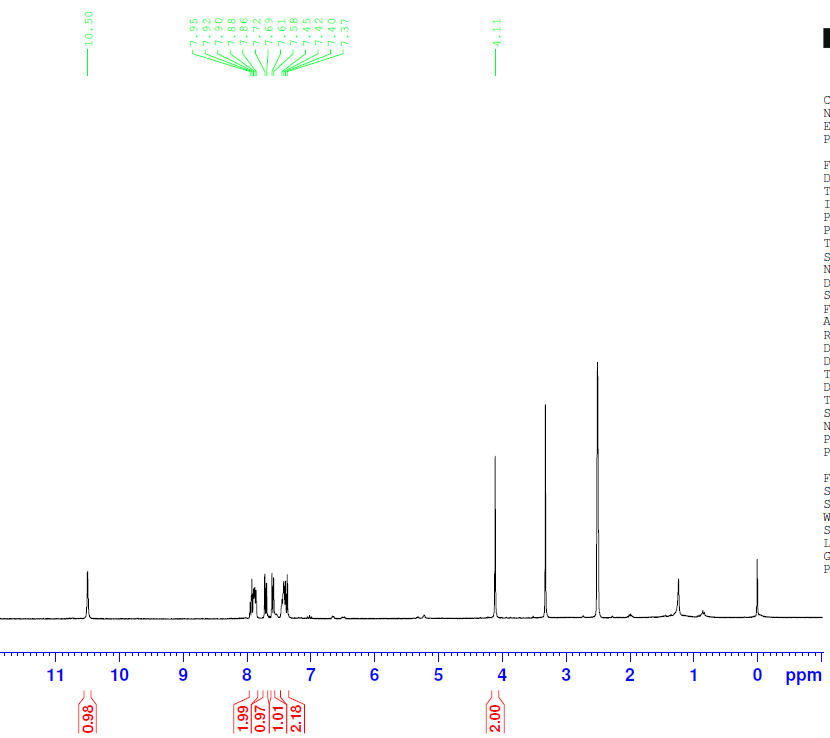


**LBJ-14**
